# The role of microtubules and the dynein/dynactin motor complex of host cells in the biogenesis of the *Coxiella burnetii*-containing vacuole

**DOI:** 10.1371/journal.pone.0209820

**Published:** 2019-01-14

**Authors:** Rodolfo M. Ortiz Flores, Jesús S. Distel, Milton O. Aguilera, Walter Berón

**Affiliations:** Instituto de Histología y Embriología, Facultad de Ciencias Médicas, Universidad Nacional de Cuyo—CONICET, Mendoza, Argentina; University of Arkansas for Medical Sciences, UNITED STATES

## Abstract

Microtubules (Mts) are dynamic cytoskeleton structures that play a key role in vesicular transport. The Mts-mediated transport depends on motor proteins named kinesins and the dynein/dynactin motor complex. The Rab7 adapter protein FYCO1 controls the anterograde transport of the endocytic compartments through the interaction with the kinesin KIF5. Rab7 and its partner RILP induce the recruitment of dynein/dynactin to late endosomes regulating its retrograde transport to the perinuclear area to fuse with lysosomes. The late endosomal-lysosomal fusion is regulated by the HOPS complex through its interaction with RILP and the GTPase Arl8. *Coxiella burnetii* (*Cb*), the causative agent of Q fever, is an obligate intracellular pathogen, which generates a large compartment with autophagolysosomal characteristics named *Cb*-containing vacuole (CCV). The CCV forms through homotypic fusion between small non-replicative CCVs (nrCCV) and through heterotypic fusion with other compartments, such as endosomes and lysosomes. In this work, we characterise the role of Mts, motor proteins, RILP/Rab7 and Arl8 on the CCV biogenesis. The formation of the CCV was affected when either the dynamics and/or the acetylation state of Mts were modified. Similarly, the overexpression of the dynactin subunit non-functional mutants p150^Glued^ and RILP led to the formation of small nrCCVs. This phenomenon is not observed in cells overexpressing WT proteins, the motor KIF5 or its interacting protein FYCO1. The formation of the CCV was normal in infected cells that overexpressed Arl8 alone or together with hVps41 (a HOPS subunit) or in cells co-overexpressing hVps41 and RILP. The dominant negative mutant of Arl8 and the non-functional hVps41 inhibited the formation of the CCV. When the formation of CCV was affected, the bacterial multiplication diminished. Our results suggest that nrCCVs recruit the molecular machinery that regulate the Mts-dependent retrograde transport, Rab7/RILP and the dynein/dynactin system, as well as the tethering processes such as HOPS complex and Arl8 to finally originate the CCV where *C*. *burnetii* multiplies.

## Introduction

*Coxiella burnetii*, a Gram-negative intracellular bacterium, is the etiological agent of human Q fever, a disease that generally manifests as an acute, debilitating flu-like illness [1]. The bacterium can survive long periods in the environment since it is highly resistant to heat, drying, and common disinfectants. *C*. *burnetii* can infect mainly monocytes/macrophages and a wide variety of host cells *in vitro* [2]. Depending on the lipopolysaccharide (LPS) content, *C*. *burnetii* presents two phase variants: the virulent Nine Mile phase I variant (NMI) and the avirulent Nine Mile phase II variant (NMII). *C*. *burnetii* phase I produces a full-length LPS, while *C*. *burnetii* phase II displays a truncated LPS [3,4]. Even though phase I and phase II *C*. *burnetii* contain LPSs of different in lengths, the intracellular behavior of both phases is similar.

*C*. *burnetii* is internalised and sequestered in small vacuoles that progressively fuse with each other and mature to generate a large vacuole named *Coxiella burnetii*-containing vacuole (CCV) [5–7]. The CCV presents autophagolysosomal characteristics, which favour bacterial replication. The CCV is highly fusogenic with different compartments of the endocytic, phagocytic and autophagic pathways and also with vesicles derived from the endoplasmic reticulum (ER) [8,9].

Mts serve as tracks for vesicular traffic in phagosome maturation. Besides, during certain infections, the Mts dynamics can be modified and controlled by the pathogen, such as *Shigella* spp and *E*. *coli* [10,11].

Mts are polar structures with two distinct ends: a fast-growing plus end and a slow-growing minus end. There are two types of Mts-based molecular motors in the cell: kinesins and the dynein/dynactin complex. Members of the kinesin family typically transport cargoes toward the plus end of Mts; by contrast, members of the dynein family do so toward the minus end of Mts [12]. Mts, and particularly dynein, are known to be involved in the invasion by several pathogens such as *Shigella* spp. and *Campylobacter jejuni* [13,14].

The stability and dynamics of Mts depend on post-translational modifications of tubulin, including detyrosination/tyrosination, acetylation/deacetylation, phosphorylation, glutamylation, glycosylation and the generation of non-tyrosinatable α-tubulin [15–17]. The acetylation/deacetylation state of tubulin has been associated with cell motility, intracellular transport and ciliary assembly/disassembly [16]. HDAC6 and αTAT are the main regulators of α-tubulin deacetylation and acetylation, respectively [18]. It has recently been demonstrated that histone deacetylase 6 (HDAC6) and NAD-dependent tubulin deacetylase sirtuin-2 (SIRT2) drive the ciliary disassembly [19] and the mitotic progression in the normal cell cycle [20]. αTAT is the main tubulin acetyltransferase in mammals [21–23]. In mice, this acetyltransferase is involved in sperm motility and fertility [24]. αTAT also participates in cell adhesion and contact inhibition of proliferation [25]. Acetylation-deacetylation modification also seems to regulate the interaction of the motors with the Mts surface [26–28].

The later steps of endosomal trafficking are under the control of the GTPase Rab7 [29]. Rab7 orchestrate the molecular machinery that controls transport, aggregation, and fusion of late endosomes and lysosomes. [30]. Interestingly, some pathogens reside in phagosomes that exclude Rab7 from their membranes [31–33], whereas others reside in phagosomes that recruit this GTPase [34]. The plus-end movement of endosomes along Mts is mediated by the interaction of Rab7 with the FYCO1 protein, which can interact with kinesin motor proteins [35]. RILP is involved in targeting the dynein-dynactin motor complex to Rab7-containing organelles [36]. RILP also regulates the recruitment of the HOPS complex (homotypic fusion and protein sorting) to endocytic compartments; it is a complex that stimulates tethering and fusion of late endosomes. HOPS is a conserved protein complex consisting of several VPS (vacuolar protein sorting) protein subunits including Vps11, Vps16, Vps18, Vps33, Vps39, and Vps41 [37–40]. The N-terminal region of RILP interacts with the HOPS complex, mainly with the C-terminal region of the Vps41 subunit [41].

Lysosomes are dynamic organelles which not only participate in the cell substrate degradation but they also play critical roles in processes such as cholesterol homeostasis, repair of the plasma membrane, antigen presentation and cell migration [42]. Arl8, which is a member of Arf-like (Arl) GTPases, has recently been identified as a crucial regulator of membrane traffic toward lysosomes and lysosome positioning. This protein mediates the kinesin I-dependent lysosome motility along the Mts towards the cell periphery [43,44]. Furthermore, Arl8 regulates membrane traffic to the lysosomes through the recruitment of HOPS complex subunits. The Vps41-Arl8 interaction regulates the endocytic cargo degradation. It has been proposed that Arl8-positive lysosomes and Rab7-positive late endosomes fuse through interaction with the HOPS complex [45].

Many pathogens can modulate the activity of Rab GTPases through the secretion of effectors into the host cell cytoplasm [46]. For example, *Mycobacterium tuberculosis* and *Listeria monocytogenes* have been found in modified Rab5-positive endocytic compartments [47]. *Tropheryma whipplei* resides in a Rab5-Rab7 positive phagocytic compartment that does not fuse with lysosomes. *Burkholderia cenocepacia* can survive within macrophages because it arrests the fusion of phagosomes with lysosomes by acting at the level of Rab7 function [48]. The CCV is intensely labelled with Rab7, and this GTPase is known to regulate the vacuole biogenesis [5].

In the present work, we evaluate the role of Mts, the acetylation of tubulin, motor complexes and GTPases involved in late endolysosomal trafficking and tethering process in the biogenesis of the CCV. Through the treatment of *C*. *burnetii*-infected HeLa cells with either nocodazole or taxol, or the overexpression of the deacetylases HDAC6 and SIRT2 and αTAT, we demonstrated the crucial role of the dynamics of Mts in the biogenesis of the CCV. Unlike the overexpression of the dynein complex, the overexpression of KIF5 inhibited the formation of the CCV. Furthermore, the overexpression of Rab7, RILP, and Arl8 allowed the formation of the CCV through the interaction with motor proteins and the HOPS complex. These findings would suggest that after internalization, *C*. *burnetii* travels on Mts inside small vacuoles containing bacteria (non-replicative CCVs, nrCCVs), using the dynein/dynactin complex to move in a retrograde manner, while acquiring the tethering molecular machinery to fuse with each other, with endosomes and lysosomes to form the characteristic CCV where bacteria replicate.

## Material and methods

### Materials

Dulbecco’s Modified Eagle's Medium (D-MEM), fetal bovine serum (FBS), penicillin and streptomycin were obtained from Gibco BRL/Life Technologies (Buenos Aires, Argentina). Plasmids encoding EGFP-HDAC6 WT and EGFP-HDAC6 H216A/H611A were kindly provided by Francisco Sánchez Madrid (Instituto de Investigación Sanitaria Princesa IIS-IP, Universidad Autónoma de Madrid, Spain). Plasmids encoding EGFP-αTAT WT and EGFP-αTAT D157N were kindly provided by Philippe Chavrier (Intitut Curie, Paris, France). Plasmids encoding EGFP-p150^Glued^WT, EGFP-p50d^ynamitin^WT and DsRed-p150^Glued^CC1 were kindly provided by Jean Celli (Laboratory of Intracellular Parasites, Rocky Mountain Laboratories, National Institute of Allergy and Infectious Diseases, National Institutes of Health, Hamilton, USA). Plasmids encoding DsRed-RILP WT and DsRed-RILP ΔN were kindly provided by Jacques Neefjes (Nederlands Kanker Instituut, Amsterdam, Netherlands). Plasmids encoding EGFP-KIF5B WT and EGFP-KIF5B 332–963 were kindly provided by Juan Bonifacino (National Institutes of Health, Bethesda, USA). Plasmids encoding EGFP-FYCO1 WT and EGFP-FYCO1 Δ555–1136 were kindly provided by Terje Johansen (Institute of Medical Biology, University of Tromsø, Tromsø, Norway). Plasmids encoding HA-hVps41 WT and HA-hVps41 A187T were kindly provided by Mahak Sharma (Indian Institute of Science, Education and Research Mohali, Punyab, India). Plasmids encoding EGFP-Arl8 WT and EGFP-Arl8 T34N were kindly provided by Roberto Botelho (Department of Chemistry and Biology, Ryerson University, Toronto, ON). Plasmids encoding HA-SIRT2 WT and HA-SIRT2 NLSΔNES were kindly provided by Bernhard Lüscher (Uniklinik RWTH Aachen, Institut für Biochemie und Molekularbiologie, Aachen, Germany). Plasmids encoding pEGFP-Rab7 WT, pEGFP-Rab7 T22N and pEGFP-Rab7 Q67L were kindly provided by Bo van Deurs (University of Copenhagen, Copenhagen, Denmark).

The rabbit polyclonal anti-*Coxiella burnetii* serum was kindly provided by Robert Heinzen (Rocky Mountain Laboratories, NIAID, NIH, Hamilton, MTS, USA). The anti-HA monoclonal antibody was obtained from Sigma-Aldrich (Argentina). The monoclonal antibody against α-tubulin was kindly provided by Cristian Acosta (IHEM, CONICET). Secondary antibodies were purchased from Jackson ImmunoResearch Laboratories, Inc. (West Grove, PA, USA Phalloidin). Taxol and nocodazole were obtained from Sigma-Aldrich (Argentina).

### Cell culture

HeLa and Vero cells (Asociación Banco Argentino de Células, Buenos Aires, Argentina) were grown in DMEM supplemented with 10% heat-inactivated FBS, 2.2 g/l sodium bicarbonate, 2 mM glutamine, 100 IU/ml penicillin and 100 μg/ml streptomycin, pH 7, at 37°C under a 5% CO_2_ atmosphere.

### Propagation of phase II *C*. *burnetii*

Clone 4 phase II Nine Mile strain of *C*. *burnetii*, which is infective for cells in culture but not for animals, was provided by Ted Hackstadt (Rocky Mountain Laboratories, NIAID, NIH, Hamilton, MTS, USA) and handled in a biosafety level II facility. Non-confluent Vero cells were cultured in T25 flasks at 37°C under a 5% CO_2_ atmosphere in DMEM supplemented with 5% FBS, 0.22 g/l sodium bicarbonate and 20 mM Hepes, pH 7 (MfbH). Cultures were infected with *C*. *burnetii* phase II suspensions for 6 days at 37°C under a 5% CO_2_ atmosphere. To prepare cell lysates, cells were frozen at -70°C, then thawed at 37°C, scraped and passed 20 times through a 27 μm gauge needle connected to a syringe. Cell lysates were centrifuged at 800 x *g* for 10 min at 4°C. Supernatants were centrifuged at 24,000 x *g* for 30 min at 4°C, and pellets containing *C*. *burnetii* were resuspended in phosphate-buffered saline (PBS; 10 mM sodium phosphate, 0.9% NaCl), aliquoted and frozen at -70°C.

### Infection of HeLa cells with *C*. *burnetii*

A total of 0.5x10^5^ HeLa cells were seeded on sterile glass coverslips placed in 24-well plates and grown overnight (see above). For infection, a 1μl aliquot of *C*. *burnetii* suspension was added to each well (multiplicity of infection: 20–40). Cells were incubated overnight at 37°C under 5% CO_2_ for bacterial internalization. After that, cells were post-incubated for 48 h to allow the formation of *C*. *burnetii*-containing vacuole.

### Treatment of infected cells with taxol and nocodazole during the formation of *C*. *burnetii* containing vacuole

Infected HeLa cells were treated with taxol (2 μM) or nocodazole (2 μM) during 48 h post-infection at 37°C under a 5% CO_2_ atmosphere.

### Fluorescence staining

HeLa cells were fixed with 4% paraformaldehyde solution in PBS for 10 min at 37°C, washed with PBS, and blocked with PBS with 5% SFB. Subsequently, cells were permeabilized with 0.05% saponin in PBS containing 0.5% BSA and then incubated with primary antibodies against *C*. *burnetii* (1:800). After washing, cells were incubated with secondary antibodies conjugated to Cy2, Cy3 or Cy5 (1:500). To detect the HA-tag, antibodies conjugated to Alexa Fluor 488 and Cy3 were used. Cells were mounted with Mowiol and examined by fluorescence microscopy

### Cell transfection

Infected cells were transfected for 2 h with 1 μg/ml of an empty pEGFP vector, pEGFP encoding HDAC6 WT, HDAC6 H216A/H611A, αTAT WT, αTAT D157N, KIF5B WT, KIF5B 332–963, FYCO1 WT, FYCO1 Δ555–1136, p150^Glued^WT, p50^dynamitin^WT, Arl8 WT, Arl8 T34N, Rab7 WT, Rab7 T22N and Rab7 Q67L, DsRed encoding RILP WT, RILP ΔN and p150^Glued^CC1 or HA encoding hVps41 WT, hVps41 A187T, SIRT2 WT and SIRT2 NLSΔNES using the Lipofectamine 2000 reagent according to the manufacturer’s instructions (Invitrogen, Buenos Aires, Argentina).

### Foci-forming unit (FFU) assay

This assay was performed according to Howe *et al*. [9]. Briefly, a total 1,5x10^5^ Vero cells were seeded on sterile glass coverslips placed in 24-well plates and incubated overnight to reach confluency. Infected cells subjected to different experimental conditions were lysed with hypotonic buffer and then scrapped from a six-well plate. Ten-fold serial dilutions of the cell lyses in cultured medium were used to infect Vero cells. After incubating 16 h at 37°C under a 5% CO_2_ atmosphere, cells were washed with PBS and incubated in culture medium for 48 h. Cells were fixed in methanol and the fluorescent staining of infectious foci was performed by indirect immunofluorescence using an anti-*C*. *burnetii* antibody and an Alexa Fluor 488-conjugated goat anti-rabbit IgG as secondary antibody (Molecular Probes cat # A-11034). FFU were quantified in 10 fields of each sample using a Nikon Eclipse TE2000 microscope with a 20x objective.

### Determination of size and number of *C*. *burnetii*-containing vacuoles

Infected cells were defined as those containing at least one bacterium inside, detected by immunofluorescence. An average of 50 cells per coverslip was calculated (in triplicate) to determine the diameter and number of vacuoles containing *C*. *burnetii*. Images were acquired with a Nikon Eclipse TE2000 microscope with a 60x objective, and analysed by phase contrast microscopy and assumptions with the fluorescence image to be able to observe the limit of the CCV correctly (and the location of the fluorescent protein overexpressed in relation to the CCV). The size and number of CCV were calculated by means of a morphometric analysis using the different measurement tools of the ImageJ software.

### Fluorescence microscopy

HeLa cells were analysed under an Eclipse TE2000 inverted microscope (Nikon, Japan). Images were obtained with a charge-coupled device camera (Orca I; Hamamatsu) and processed with the Metamorph 6.1 software (Universal Images Corporation). Representative images of each experiment were acquired with the Olympus FV1000 confocal microscope and the FV 10-ASW 1.7 software (Olympus, Japan). Images were deconvoluted using the ImageJ software (NIH [http://rsb.info.nih.gov/ij]). The degree of co-localization between CCV and the proteins of interest was quantified in control and infected cells. The localization degree of the proteins under study with the CCV in phase contrast images was analysed by the Pearson coefficient. The correlation of fluorescent intensity to quantify co-localization of two proteins on the CCV was analysed by the Manders coefficient. Proteins were considered to co-localize when the values of the coefficients were above 0.5. The analysis was done using the JACoP plugin (Just Another Co-localization Plugin; NIH [https://imagej.nih.gov/ij/plugins/track/jacop2.html]) of the ImageJ software.

### Statistical analysis

Data were expressed as the means ± standard error of the mean (SE) from three independent experiments. Statistics were performed with the GraphPad Prism software using one-way ANOVA and/or Student’s two-tailed *t*-test followed by the Tukey’s comparisons test.

## Results

### Host cell microtubules-dependent biogenesis of the *C*. *burnetii*-containing vacuole

With the aid of molecular motors, Mts are used as structural support and as tracks to guide and transport intracellular cargoes [49]. Both the cargo transport and the Mts assembly and/or disassembly can be used for certain intracellular pathogens [50]. To determine if any modification occurring at the Mts level affects the biogenesis of the *C*. *burnetii*-containing vacuole (CCV), we assessed the effect of taxol and nocodazole during a cell post-infection period. We first determined, by Trypan Blue staining, if the treatment with these drugs affected cell viability. As shown in S1 Fig, the incubation for different times with taxol or nocodazole (2 μM) did not affect cell viability.

**Fig 1:**
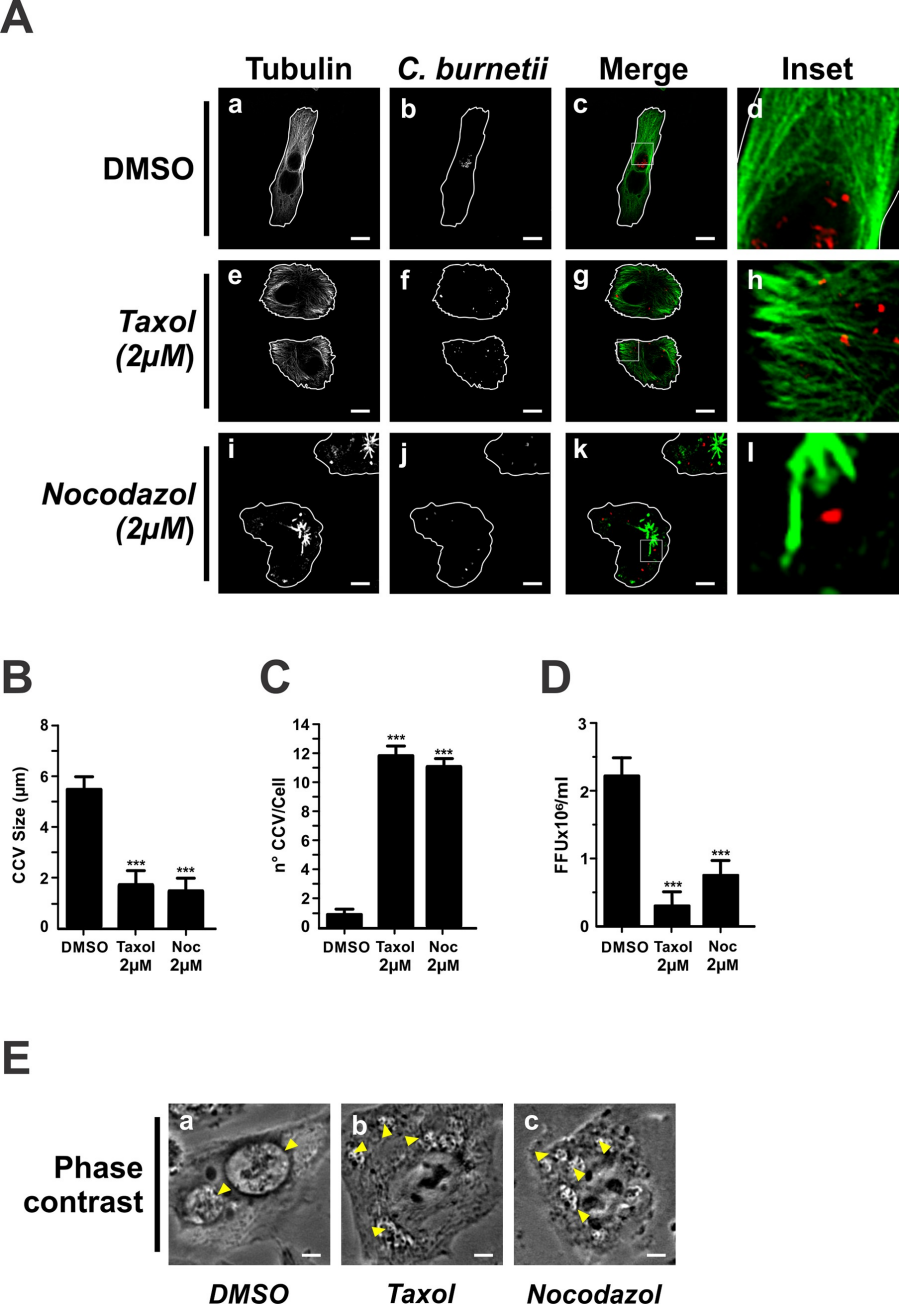
Dynamic microtubules participate in the formation of the CCV. HeLa cells were infected for 16 h with *Coxiella burnetii* and incubated for 48 h with either DMSO (control), taxol or nocodazole. Cells were then fixed and processed for IIF. Mts were labelled with an anti-α tubulin antibody (green pseudo-colour) or *C*. *burnetii* with a specific antibody (red pseudo-colour). (A) Cells treated with 0.1% DMSO (panel a-d), 2 μM taxol (panel e-h), and 2 μM nocodazole (Noc) (panel i-l). Scale bar: 10 μm. Quantitative analysis of CCV size (B) and number (C)s, and bacterial multiplication (D). Forty to sixty cells were analysed in each experiment. Results are expressed as means ± SE of three independent experiments. ***p<0.001. (E) Phase contrast microscopy of infected and transfected HeLa cells. Arrowheads indicate a nrCCV (panel b and c), or a CCV (panel a). Scale bar: 2 μm.

HeLa cells were infected for 16 h and then incubated for 48 h with either taxol or nocodazole. After incubating, cells were processed for IIF and analysed by confocal microscopy. To distinguish the compartments that contain *C*. *burnetii*, cells were analysed by phase-contrast bright-field microscopy. When the Mts were altered by taxol or nocodazole, *C*. *burnetii* was found in small vacuoles. As a rule, these vacuoles contained a single bacterium, suggesting a low bacterial replication rate (Fig 1A, panels f and j, E panels b and c). The size of these (1.76±0.32 μm and 1.52±0.55 μm, for vacuoles altered by taxol and nocodazole, respectively) was around four folds smaller than the CCV size observed in DMSO-treated cells (5.48±0.61 μm) (Fig 1A and 1B). While one CCV was observed in control cells incubated with DMSO (Fig 1A, panel b, and 1B), approximately twelve nrCCVs were observed in treated cells (Fig 1 A, panels f and j, and C). To determine if the phenotype alteration caused by the treatment with either taxol or nocodazole alters bacterial replication, intracellular bacteria were quantified by the foci-forming unit (FFU) assay. Bacterial replication was diminished by 84% in cells treated with taxol and by 67% in cells treated with nocodazole, as compared to control cells incubated with DMSO (Fig 1D). The decrease in bacterial multiplication would be in agreement with the phenotype displayed by small vacuoles containing a single bacterium. These results suggest that the formation of the CCV and bacterium multiplication requires dynamic Mts.

**Fig 2:**
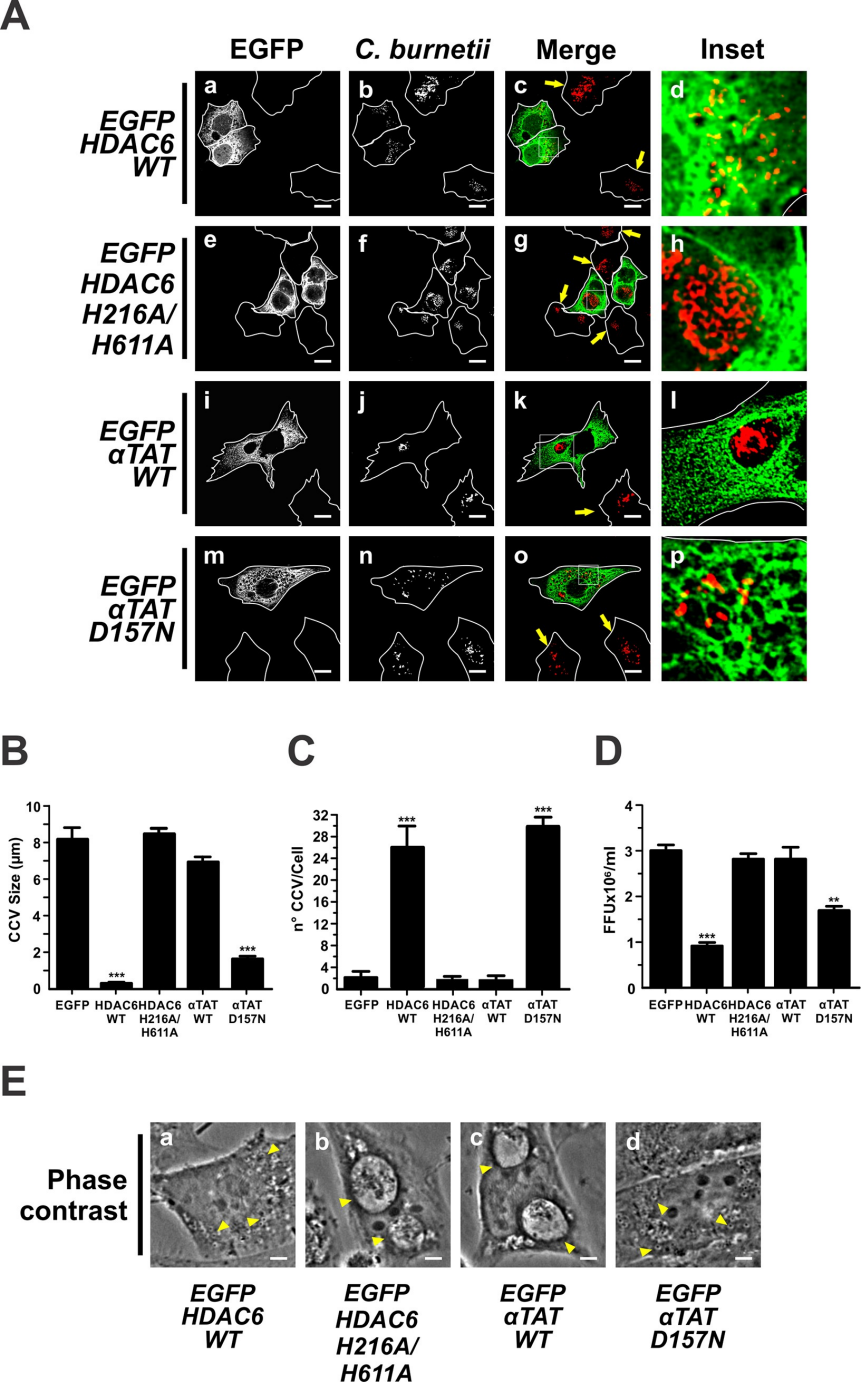
Overexpression of HDAC6 and αTAT regulate the formation of CCV. (A) Infected HeLa cells were transfected with pEGFP-HDAC6WT (panels a-d), -HDAC6 H216A/H611A (panels e-h), -αTAT WT (panels i-l) or -αTAT D157N (panels m-p). Cells were fixed and processed for IIF. An anti-*C*. *burnetii* (red pseudo-colour) antiserum was used for detecting bacteria. Arrows indicate untransfected cells containing CCVs. Scale bar: 10 μm. Quantitative analysis of CCV size (B), number (C), and bacterial multiplication (D). Forty to sixty cells were analysed in each experiment. Results are expressed as means ± SE of three independent experiments. **p<0.01, ***p<0.001. (E) Phase contrast microscopy of infected and transfected HeLa cells. Arrowheads indicate a nrCCV (panel a and d), and a CCV (panel b and c). Scale bar: 2 μm.

Although controversial, the Mts stability has been related to post-translational modifications of α-tubulin, such as the acetylation-deacetylation [51–53]. Deacetylation is known to be carried out by HDAC6 and SIRT2, while acetylation is catalysed by αTAT [54]. To determine whether the HDAC6 and SIRT2 were related to the biogenesis of the CCV, HeLa cells were infected with *C*. *burnetii* and then transfected with plasmids encoding either HDAC6 WT (wild type) or its mutant HDAC6 H216A/H611A or αTAT WT or its mutant αTAT D157N. These mutants are catalytically inactive enzymes that cannot covalently modify Mts [55]. As shown in Fig 2A, the overexpression of HDAC6 WT prevented the formation of the CCV (panels a-d) while such process was not affected by the inactive mutant (panels e-h). Interestingly, non-transfected cells showed CCV (panels a-c, arrow pointed cells). This observation was corroborated when the size and number of CCV were determined. In cells expressing HDAC6 WT, the CCV size was 0.5±0.1 μm *vs*. 8.5±0.5 μm in cells expressing HDAC6 H216A/H611A (Fig 2B). The CCV number was 26±4 vacuoles/cell in HDAC6 WT cells *vs*. 2.0±5.0 vacuoles/cell in HDAC6 H216A/H611A cells (Fig 2C). Similar differences were observed between cells overexpressing the HDAC6 WT and control cells overexpressing EGFP (Fig 2B and 2C). The bacterial multiplication was inhibited by 73% in cells overexpressing HDAC6 WT as compared to cells overexpressing EGFP (control) and cells overexpressing HDAC6 H216A/H611A (Fig 2D).

The role of SIRT2 deacetylase was also evaluated. Infected HeLa cells were transfected with plasmids encoding HA-SIRT2 WT and its mutant HA-SIRT2 NLΔNES. This truncated mutant lacks the NES domain (nuclear export signal), therefore it cannot be transported from the nucleus to cytoplasm to catalyse substrate deacetylation [56]. As shown in S2 Fig, HA-SIRT2 WT remained dispersed in the cell cytoplasm and induced the formation of small and numerous nrCCVs (S2A Fig, panels a-d, S2B and S2C). A CCV was observed in a non-transfected cell (S2A Fig, panels a-c, arrow pointed cells). In cells overexpressing the mutant HA-SIRT2 NLΔNES, which was sequestered in the nucleus, CCVs presented size and number that was similar to those observed in control cells overexpressing EGFP (S2A Fig, panels e-h, S2B and S2C). The bacterial multiplication was inhibited by 60% in cells overexpressing HA-SIRT2 WT, as compared to that observed in EGFP overexpressing cells (control) and HA-SIRT2 NLΔNES overexpressing cells (S2D Fig).

It is expected that the overexpression of αTAT have an opposite effect to that observed with HDAC6 and SIRT2. As shown in Fig 2A, CCVs were observed not only in cells overexpressing αTAT WT (panels i-l) but also in control cells (data not shown). In contrast, the mutant αTAT D157N inhibited the formation of the CCV (panels m-p). This observation was corroborated when the size and number of CCVs were determined (αTAT WT: 7.0±0.2 μm *vs*. αTAT D157N: 1.6±0.2 μm; αTAT WT: 2.0±1.0 vacuoles/cell *vs*. αTAT D157N 30.0±2.0 vacuoles/cell; Fig 2B and 2C). Similar differences in the CCV size and number were observed in cells overexpressing the αTAT D157N, as compared to control cells overexpressing EGFP (Fig 2B and 2C). CCVs were observed in cells that did not express EGFP-αTAT D157N (Fig 2A, panels m-o, arrow pointed cells). Bacterial multiplication was inhibited by 47% in cells overexpressing αTAT D157N in comparison to cells overexpressing αTAT WT and control cells overexpressing EGFP (Fig 2D).

To check the acetylation status of Mts, HeLa cells were infected with *C*. *burnetii*, then transfected with plasmids encoding either HDAC6 WT or αTAT WT, and processed by immunofluorescence to detect acetylated α-tubulin using a specific antibody. As shown in S3 Fig, moderate levels of acetylated Mts were observed in untransfected cells (arrow pointed cells). In cells overexpressing HDAC6 WT, Mts acetylation was slightly reduced (panels a-d), as compared to non-transfected cells (arrow pointed cells). High Mts acetylation levels were observed in cells overexpressing αTAT WT (panel e-h) as compared to untransfected cells (arrow pointed cells). nrCCVs were observed in cells overexpressing HDAC6 WT (panel c) while CCVs developed in cells overexpressing αTAT (panel g) and in untransfected cells (arrow pointed cells). HDAC6 WT deacetylase mainly acts on tubulin dimers [57], which could justify the low or basal levels of Mts acetylation found in cells overexpressing HDAC6 WT. Although the formation of CCV was observed in cells overexpressing αTAT with high levels of Mts acetylation, we cannot assert that our observation can be attributed to this post-translation modification. HDAC6 and αTAT participate in the regulation of the dynamics of Mts rather than in their stability [58–61]. Taking into account the effects caused by the overexpression of these enzymes and by the treatment with either nocodazole or taxol on the formation of the CCV, we conclude that the dynamics of Mts are crucial for the biogenesis of the CCV.

### The dynein/dynactin motor complex is involved in the formation of the *C*. *burnetii*-containing vacuole

**Fig 3:**
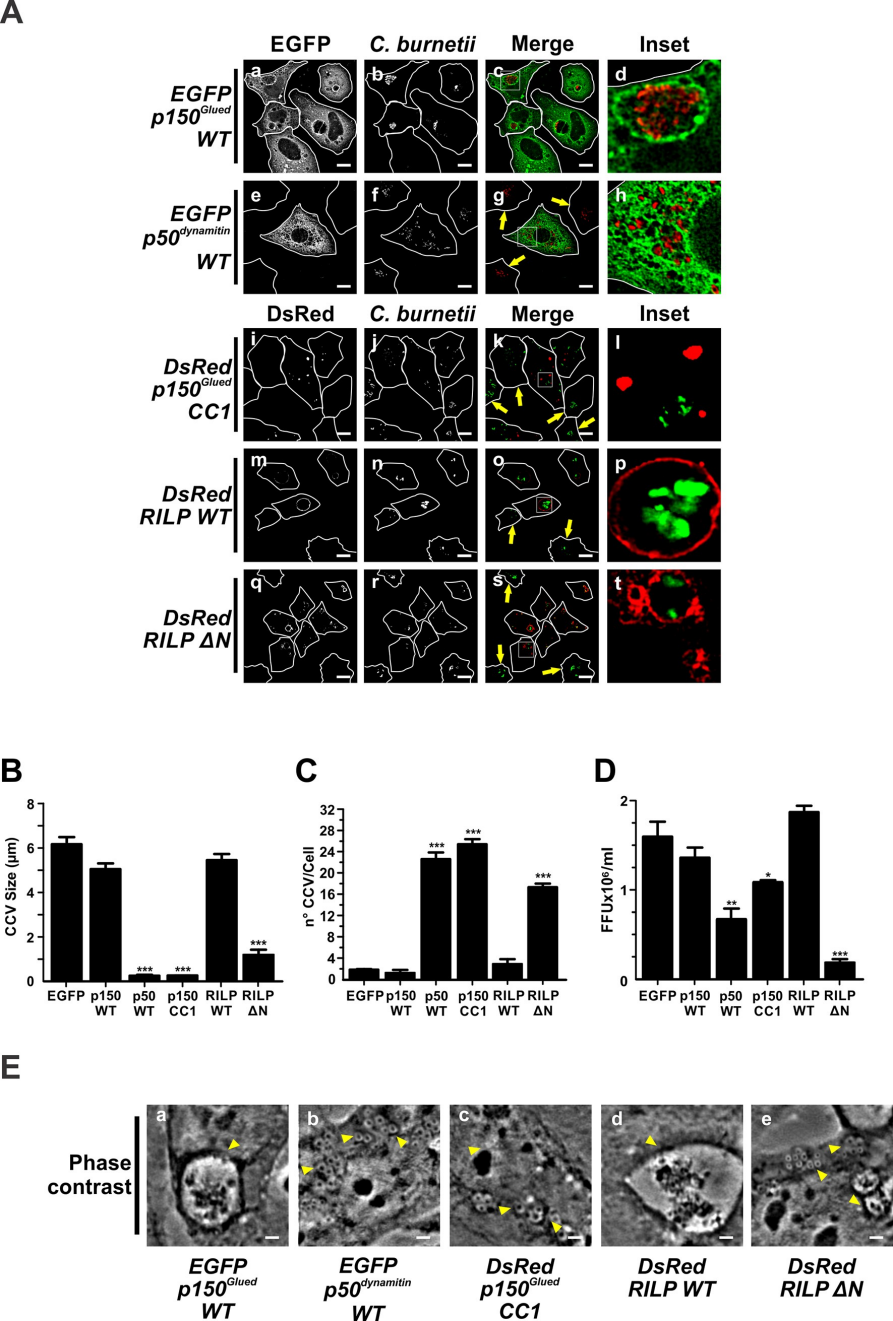
The formation of CCV is regulated by the dynein/dynactin motor complex. (A) Infected HeLa cells were transfected with pEGFP-p150^Glued^WT (panels a-d), -p50^dynamitin^WT (panels e-h), pDsRed-p150^Glued^CC1 (panels i-l), -RILP WT (panels m-p) or -RILP ΔN (panels q-t). Arrows indicate non-transfected cells containing CCV. Scale bar: 10μm. Cells were fixed and processed for IIF. *C*. *burnetii* was detected with an anti-*C*. *burnetii* antiserum (panels b-h, red pseudo-colour; panels j-t, green pseudo-colour. Quantitative analysis of CCV size (B) and number (C), and bacterial multiplication (D). Forty to sixty cells were analysed in each experiment. Results are expressed as means ± SE of three independent experiments. *p<0.05; ***p<0.001. (E) Phase contrast microscopy of infected and transfected HeLa cells. Arrowheads indicate a nrCCV (panels b, c and e), or a CCV (panels a and d). Scale bar: 2 μm.

The dynein/dynactin motor complex is responsible for the Mts-dependent intracellular retrograde transport. Dynactin is a heterocomplex comprising several subunits, including p150^Glued^, p50^dynamitin^ and Arp1 [62–64]. To determine the role of the dynein/dynactin motor complex, cells were infected and then transfected with pEGFP-p150^Glued^WT or pEGFP-p50^dynamitin^WT. As shown in Fig 3A (panels a-d) and Table 1, the overexpressed p150^Glued^WT was recruited to CCV membranes without inducing a significant modification in the size (5.0 ±0.2 μm) and number (4.0±1.0 vacuoles/cell) of CCV, as compared to control cells overexpressing EGFP (6.1±0.1 μm, 2.0±0.2 vacuoles/cell) (Fig 3B and 3C).

**Table 1:** Pearson’s and Manders’ colocalization coefficients.

Coefficients	Values
Pearson CCV—RILP WT	0.824 ± 0.021
Manders RILP WT—Rab7 WT M1	0.700 ± 0.050
Manders Rab7 WT—RILP WT M2	0.625 ± 0.005
Manders RILP WT—Rab7 T22N M1	0.175 ± 0.026
Manders Rab7 T22N - RILP WT M2	0.350 ± 0.018
Manders RILP WT—Rab7 Q67L M1	0.650 ± 0.005
Manders Rab7 Q67L - RILP WT M2	0.640 ± 0.020
Manders RILP WT—p150 WT M1	0.800 ± 0.010
Manders p150 WT—RILP WT M2	0.815 ± 0.020
Manders RILP WT—p50 WT M1	0.900 ± 0.023
Manders p50 WT—RILP WT M2	0.620 ± 0.030
Manders RILP WT—hVps41 WT M1	0.840 ± 0.016
Manders hVps41 WT—RILP WT M2	0.890 ± 0.018
Manders RILP WT—hVps41 A187T M1	0.160 ± 0.020
Manders hVps41 A187T - RILP WT M2	0.163 ± 0.022
Pearson CCV—RILP ΔN	0.710 ± 0.010
Manders RILP ΔN—Rab7 WT M1	0.700 ± 0.025
Manders Rab7 WT—RILP ΔN M2	0.725 ± 0.012
Manders RILP ΔN—p150 WT M1	0.850 ± 0.025
Manders p150 WT—RILP ΔN M2	0.650 ± 0.012
Manders RILP ΔN—hVPs41 WT M1	0.850 ± 0.020
Manders hVps41 WT—RILP ΔN M2	0.775 ± 0.025
Pearson CCV—p150 WT	0.675 ± 0.025
Pearson CCV—p150 CC1	0.100 ± 0.010
Pearson CCV—p50 WT	0.225 ± 0.025
Pearson CCV—Arl8 WT	0.860 ± 0.030
Pearson CCV—Arl8 T34N	0.350 ± 0.025
Pearson CCV—hVps41 WT	0.800 ± 0.045
Manders hVps41 WT—Arl8 WT M1	0.675 ± 0.025
Manders Arl8 WT—hVps41 WT M2	0.730 ± 0.025
Manders hVps41 WT—Arl8 T34N M1	0.900 ± 0.020
Manders Arl8 T34N - hVps41 WT M2	0.605 ± 0.022
Pearson CCV—hVps41 A187T	0.200 ± 0.025
Manders hVps41 A187T - Arl8 WT M1	0.630 ± 0.015
Manders Arl8 WT—hVps41 A187T M2	0.873 ± 0.015
Pearson CCV—FYCO1 WT	0.750 ± 0.023
Pearson CCV—FYCO1 Δ555–1136	0.800 ± 0.005

The CCV phenotype was altered in cells overexpressing the mutant p150^Glued^CC1. This mutant is known to bind dynein and to disrupt the dynein-dynactin interaction, thus altering the motor activity [65]. As shown in Fig 3A (panels i-l), nrCCVs were observed instead of a CCV. When the cells presented in panels i-k were analysed; CCVs were observed in non-transfected cells (arrow pointed cells). The quantification showed small (0.5±0.1 μm) and numerous (25.5±0.5 vacuoles/cell) nrCCVs, as compared to control cells overexpressing EGFP (Fig 3B and 3C). In cells overexpressing p150^Glued^CC1, a statistically significant inhibition (35%) of bacterial multiplication was observed, as compared to p150^Glued^WT or EGFP overexpressing cells (control) (Fig 3D).

It has been described that the overexpressed p50^dynamitin^WT subunit disrupts the dynein-dynactin motor complex dispersing p150^Glued^WT into the cytoplasm [66]. To assess whether the assembly of the dynein-dynactin complex is necessary for the formation of the CCV, infected HeLa cells were transfected with a pEGFP-p50^dynamitin^WT. As shown in Fig 3A (panels e-h), 3B and 3C, small (0.5±0.1 μm) and numerous (23.0±1.0 vacuoles/cell) nrCCVs were observed in EGFP-p50^dynamitin^WT overexpressing cells. Non-transfected cells presented a CCV (panels e-g, cells pointed by arrows). In cells overexpressing EGFP-p50^dynamitin^WT, a statistically significant inhibition (55%) in bacterial multiplication was observed, as compared to control cells overexpressing EGFP (Fig 3D). These results suggest that a functional dynein-dynactin motor complex is required for the formation of the big vacuoles that shelter *C*. *burnetii*.

There is evidence suggesting that motor proteins have more affinity for acetylated Mts [18,27,63,67,68]. The latter finding is in line with the observation that infected cells co-expressing HA-SIRT2 NLΔNES and p150^Glued^WT developed CCVs labelled with p150^Glued^WT (S4A, S4B and S4C Fig). The same phenomenon was observed for EGFP overexpressing cells (Fig 2B and 2C). Small and numerous nrCCVs were formed in cells co-expressing HA-SIRT2WT and p150^Glued^WT. These vacuoles were not decorated with p150^Glued^WT. CCVs were observed in non-transfected cells used as internal control (panels a-c and e-g, cells pointed by arrows). These results suggest that deacetylases disrupt the formation of CCVs, even in the presence of p150^Glued^WT.

We have previously reported that the GTPase Rab7 is recruited to CCV and that its active state is necessary to allow vacuole formation [5,7]. It is known that Rab7 is a key regulator of the endosome and phagosome maturation and the Mts-mediated intracellular transport [69]. Rab7 participates in the transport toward the minus or plus ends of Mts, with the direction being defined by its binding to either RILP (Rab interacting lysosomal protein) or FYCO, which interact with dynein or kinesin, respectively. The RILP-Rab7 association plays an important role in the recruitment of the dynein-dynactin motor complex to endosomal compartments during transport towards the pericentriolar region [36,70]. We hypothesise that since *C*. *burnetii* is transported inside vacuoles along the phagocytic pathway, the pathogen takes advantage of RILP to finally generate the large CCV. To test this hypothesis, infected cells were transfected with plasmids encoding RILP WT or the truncated mutant RILP ΔN, which bind Rab7 but not the motor complex. As shown in Fig 3A (panels m-p) and Table 1, the overexpressed pDsRed-RILP WT was recruited to the CCV membrane. The size (5.5±0.2 μm) and number (3.0±1.0 vacuoles/cell) of CCVs were comparable to those observed in control cells overexpressing EGFP (6.2±0.2 μm, 2.0±0.1 vacuoles/cell) (Fig 3B and 3C). The overexpression of the truncated mutant RILP ΔN generated a high number of RILP ΔN positive nrCCVs (17.5±0.5 vacuoles/cell, 1.2±0.2 μm) (Fig 3A, panels q-t, 3B and 3C, and Table 1). The overexpression of RILP WT did not significantly affect the multiplication of *C*. *burnetii* as the truncated mutant did (86% decrease) when compared to control cells overexpressing EGFP (Fig 3D).

To confirm the role of RILP in the biogenesis of the CCV, endogenous RILP and overexpressed EGFP were knocked down by specific siRNAs. Similarly, to the effects observed after the overexpression of the dominant negative mutant RILP ΔN, the depletion of the endogenous and the overexpressed EGFP-RILP proteins led to the generation of nrCCVs instead of CCV (S5 Fig).

Taken together, these results suggest that the RILP and the dynein-dynactin motor complex participate in the biogenesis of the CCV and in bacterial multiplication.

Infected HeLa cells were either transfected or co-transfected with plasmids encoding the different proteins under study. Cells were fixed, processed for IIF and analysed by confocal microscopy. The fluorescence intensity of these proteins was analysed with specific channels. Pearson’s (mono-transfection) and Manders’ (co-transfection) coefficients were calculated using the JACoP plugin of the ImageJ software. Fifty cells overexpressing the proteins were imaged in each experiment. Results are expressed as means ± SE of three independent experiments. Data were analysed by one-way ANOVA.

### The formation of *C*. *burnetii*-containing vacuole requires Rab7, RILP and the dynein/dynactin motor complex

**Fig 4:**
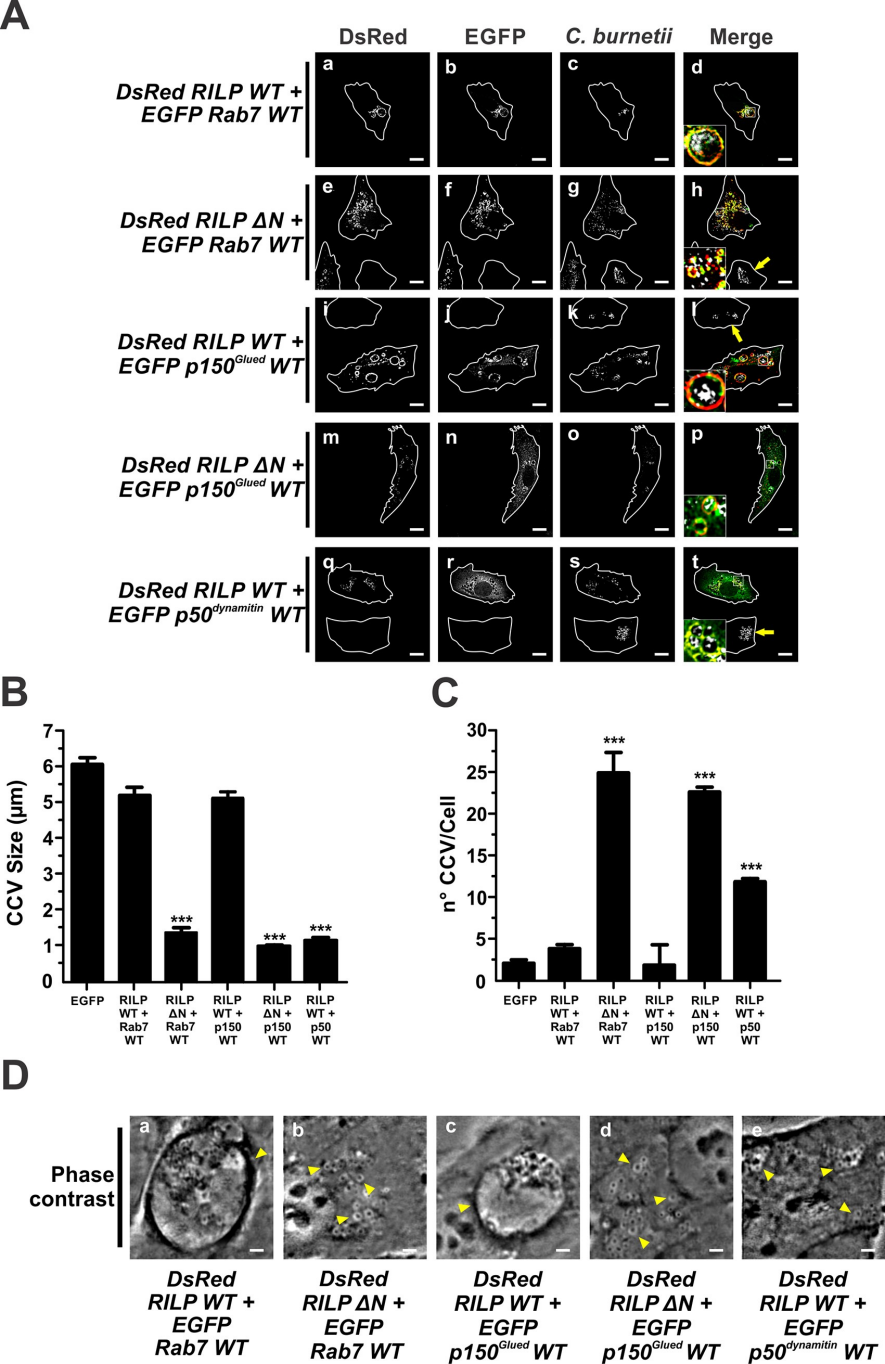
Rab 7 and its effector RILP are required for the formation of CCV. (A) Infected HeLa cells were co-transfected with pDsRed-RILP WT and pEGFP-Rab7 WT (panels a-d), pDsRed-RILP ΔN and pEGFP-Rab7 WT (panels e-h), pDsRed-RILP WT and pEGFP-p150^Glued^WT (panels i-l), pDsRed-RILP ΔN and pEGFP-p150^Glued^WT (panel m-p) or pDsRed-RILP WT and pEGFP-p50^dynamitin^WT (panels q-t). Cells were fixed and processed for IIF. *C*. *burnetii* was detected with an anti-*C*. *burnetii* antiserum (white pseudo-colour). Arrows indicate non-transfected cells containing CCV. Scale bar: 10 μm. Quantitative analysis of CCV size (B) and number (C). Forty to sixty cells were analysed in each experiment. Results are expressed as means ± SE of three independent experiments. ***p<0.001. (D) Phase contrast microscopy of infected and transfected HeLa cells. Arrowheads indicate a nrCCV (panels b, d and e), and a CCV (panels a and c). Scale bar: 2 μm.

It is known that Rab7 associates with the cytoplasmic dynein-1 through the binding of RILP to the dynactin p150^Glued^ subunit to control late endosomal transport [36]. We have previously demonstrated that Rab7 regulates the CCV biogenesis [5]. In this work, we study the role of the Rab7 effector RILP and the dynein/dynactin motor complex in that process. Cells were infected and then co-transfected with pDsRed-RILP WT/pEGFP-Rab7 WT or pDsRed-RILP ΔN/pEGFP-Rab7 WT. As shown in Fig 4A, DsRed-RILP WT and EGFP-Rab7 WT (panels a-d), and RILP ΔN and Rab7 WT (panels e-h) were recruited to CCVs (Table 1). In cells overexpressing RILP WT/Rab7 WT (Fig 4A, 4B and 4C), the size (5.2±0.3) and number (4.7±0.3) of CCVs were similar to those recorded in cells overexpressing RILP WT only (Fig 3) and control cells overexpressing EGFP (Fig 4B and 4C). On the contrary, the combination of Rab7 WT and RILP ΔN induced the formation of a higher number (25.0±2.5 vacuoles/cell) of smaller (1.6±0.1 μm) nrCCVs (Fig 4A, 4B and 4C), as compared to control cells overexpressing EGFP (Fig 4B and 4C). A similar effect was observed in cells overexpressing RILP ΔN alone (Fig 3). Interestingly, in Fig 4A (panels e-h, cells pointed by arrows), a CCV can be observed in a non-transfected cell.

In infected cells co-overexpressing DsRed-RILP WT and pEGFP-Rab7 Q67L (constitutively active mutant), the number and size of CCVs (S6 Fig) were similar to those observed in control cells overexpressing EGFP alone. Both proteins were recruited to the CCV (Table 1). In infected cells that co-overexpressed pDsRed-RILP WT and EGFP-Rab7 T22N (dominant negative mutant), the size and number of vacuoles (S6 Fig) were diminished and increased, respectively, as compared to control cells overexpressing only EGFP. Neither pDsRed-RILP WT nor EGFP-Rab7 T22N was significantly recruited to vacuoles (Table 1). The latter finding is in agreement with the regulation exerted by Rab7 on RILP [70].

To test the motor complex recruitment to the CCV membrane mediated by RILP, and the impact in the formation of the CCV, infected cells were co-transfected with either pDsRed-RILP WT or -RILP ΔN and the motor subunits pEGFP-p150^Glued^WT or -p50^dynamitin^WT. As shown in Fig 4A (panel i-l) and Table 1, both p150^Glued^WT and RILP WT decorated the CCV membranes. The combination RILP WT/p150^Glued^WT (Fig 4B and 4C) did not affect the size and number of CCVs, parameters that were similar to those of cells overexpressing RILP WT or p150^Glued^WT alone (Fig 3A–3C). In cells co-expressing DsRed-RILP ΔN and EGFP-p150^Glued^WT, RILP ΔN was found to be associated to nrCCVs (Fig 4A, panels m-p, 4B and 4C), similarly to that observed in cells expressing RILP ΔN alone (Fig 3A–3C). Interestingly, p150^Glued^WT, which was co-expressed with RILP ΔN, was found to be associated in a low degree to nrCCVs (Fig 4A, panels m-p, and Table 1). As shown above, a recruitment of overexpressed p150^Glued^WT was observed in a CCV formed in mono-transfected cells (Fig 3A–3C and Table 1).

Unlike control cells overexpressing EGFP (Fig 4B and 4C) or cells expressing RILP alone (Fig 3B and 3C), cells co-expressing RILP WT/p50^dynamitin^WT (Fig 4A, 4B and 4C), displayed several nrCCVs, (12.2 ±0.2 vacuoles/cell) with small size (1.2 ±0.2 μm). Interestingly, when the cells of panels p-t were analysed, non-transfected cells (Fig 4A, panels q-t) showed a normal CCV phenotype. The overexpression of RILP WT seemed to induce a partial reversal in the changes (size and number) exerted by the expression of p50^dynamitin^ alone (Fig 3A–3C). Interestingly, some p50^dynamitin^ co-localized to vacuoles labelled with RILP WT (Fig 4A, panels q-t, Fig 3A, panels e-h, and Table 1).

Together, these results suggest that the RILP/Rab7 association plays an important role in recruiting the dynein-dynactin motor complex to vacuoles that contain *C*. *burnetii*.

### KIF5 and FYCO1 inhibit the formation of the *C*. *burnetii*-containing vacuole

Kinesins are motors that transport cargoes toward the plus end of Mts [71]. Kinesin I (KIF5) is involved in Mts plus end transport of late endosomes [72]. FYCO1 participates actively in the anterograde cargo transport by linking Rab7 to kinesin motor proteins.

**Fig 5:**
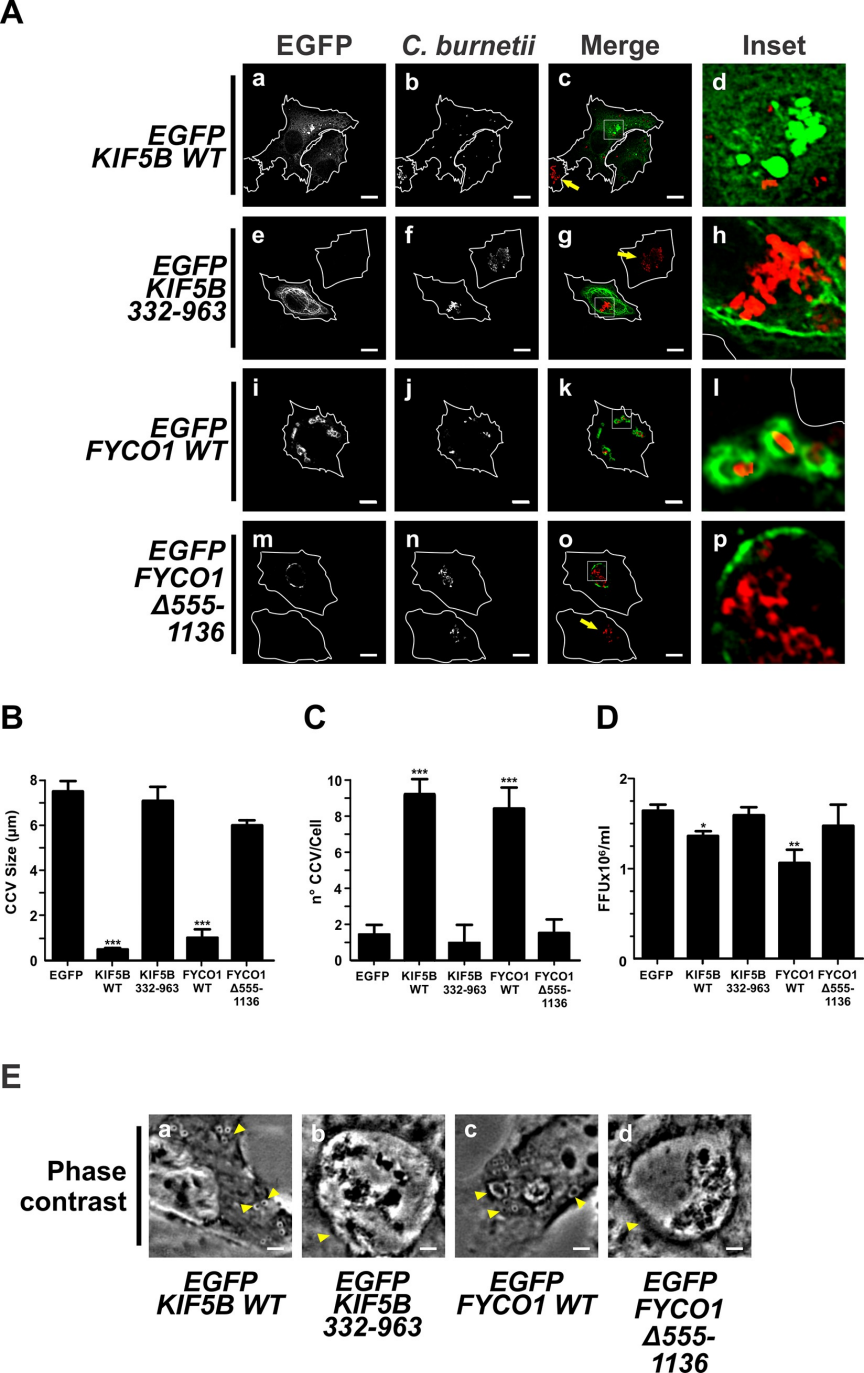
The overexpression of KIF5B and FYCO1 inhibits the formation of the CCV. (A) Infected HeLa cells were transfected with EGFP-KIF5B WT (panels a-d), or -KIF5 332–963 (panels e-h) or transfected with pEGFP-FYCO1 WT (panels i-l) or -FYCO1 Δ555–1136 (panels m-p). Cells were fixed and processed for IIF. An anti-*C*. *burnetii* antiserum was used for detecting the bacteria (red pseudo colour). Arrows indicate non-transfected cell containing CCV. Scale bar: 10 μm. Quantitative analysis of CCV size (B) and number (C) and bacterial multiplication (D). Forty to sixty cells were analysed in each experiment. Results are expressed as means ± SE of three independent experiments. *p<0.05; ** p<0.01; *** p<0.001. (E) Phase contrast microscopy of infected and transfected HeLa cells. Arrowheads indicate a nrCCV (panels a and c), or a CCV (panels b and d). Scale bar: 2 μm.

Infected cells transfected with either EGFP-KIF5B WT or EGFP-KIF5B 332–963, a motor-less form having a dominant-negative effect, were used as an experimental strategy to study the role of kinesin in the formation of the CCV. It has been demonstrated that the KIF5B 332–963 mutant causes a juxtanuclear clustering of lysosomes [73]. Infected cells overexpressing EGFP-KIF5B WT presented a higher number (9.0±1 vacuoles/cell) and a smaller size (1.5±0.2 μm) of vacuoles containing *C*. *burnetii* (Fig 5A, panels a-d, 5B and 5C) than those observed in cells overexpressing EGFP-KIF5B 332–963 (1.0±1.0 vacuoles/cell; 7.0±0.5 μm) (Fig 5A, panels e-h, 5B and 5C), or control cells overexpressing EGFP (1.5±0.5 vacuoles/cell; 8.5±0.5 μm) (Fig 5B and 5C). The multiplication rate of *C*. *burnetii* was inhibited by 22% in cells overexpressing EGFP-KIF5B WT, when compared to control cells overexpressing EGFP alone or cells overexpressing EGFP-KIF5B 332–963 (Fig 5D).

FYCO is an effector of Rab7 and an adaptor for kinesin, which participates in phagosome tubulation processes [35] and autophagosome trafficking [74]. To study the possible relationship between FYCO and the formation of CCV, infected cells were transfected with pEGFP-FYCO1 WT or the pEGFP-FYCO1 Δ555–1136 mutant. As shown in Fig 5A both overexpressed constructs EGFP-FYCO1 WT (panels i-l) and its non-functional mutant EGFP-FYCO1 Δ555–1136 (panels m-t), were recruited to the membrane of vesicles containing *C*. *burnetii* (Table 1). It is known that the construct EGFP-FYCO1 Δ555–1136 binds to Rab7 but not to the kinesin motor [74]. In cells overexpressing EGFP- FYCO1 WT, the sizes of compartments containing *C*. *burnetii* decreased (0.5±0.1 μm) while its number increased (4.2±0.1 vacuoles/cell), as compared to cells overexpressing EGFP-FYCO1 Δ555–1136 (6.0±0.2 μm, 0.7±0.3 vacuoles/cell) or to control cells overexpressing EGFP (Fig 5B and 5C). The multiplication rate of *C*. *burnetii* was not affected by the overexpression of EGFP-FYCO1 Δ555–1136; however, a significant decrease (37%) was observed in cells overexpressing FYCO1 WT, as compared to control cells overexpressing EGFP (Fig 5D).

These results suggest that *C*. *burnetii* resides in a compartment formed under the regulation of FYCO1 and KIF5.

### The HOPS complex participates in the formation of the *C*. *burnetii*-containing vacuole through the interaction with RILP and Arl8

HOPS (homotypic fusion and protein sorting) is a complex that plays a critical role in regulating the late stage of the endocytic pathway by driving the late endosomal membrane tethering and fusion. This complex consists of several subunits, in particular, Vps39 and Vps41 are subunits that presumably interact with Rab7, and can bind RILP [75]. The Vps41 subunit is required for the stabilization of the HOPS complex [38,76].

Knowing that the formation of the CCV involves vesicle fusion and that the CCV is highly fusogenic [77], HOPS is expected to participate in the CCV biogenesis. To study the role of the HOPS complex in the formation of the CCV, infected cells were transfected with plasmids encoding HA-hVps41 WT or its mutant HA-hVps41 A187T, which cannot bind RILP [78].

**Fig 6:**
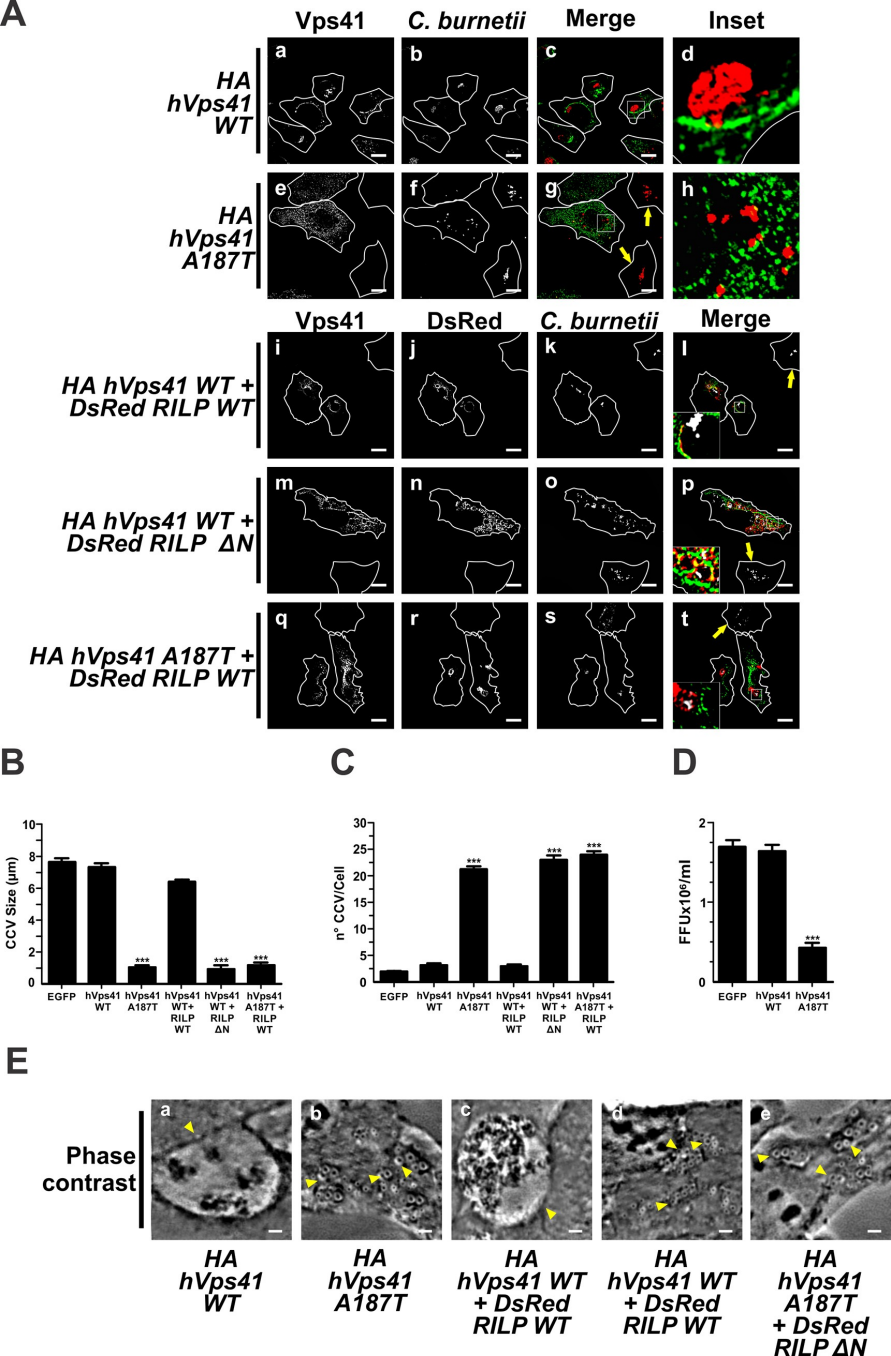
HOPS and RILP participate in the development of the CCV. (A) Infected HeLa cells were transfected with plasmids encoding HA-hVps41 WT (panels a-d) or -hVps41 A187T (panels e-h) or co-transfected with plasmids encoding HA-hVps41WT and DsRed-RILP WT (panels i-l), HA-hVps41WT and DsRed-RILPΔN (panels m-p) or HA-hVps41 A187T and DsRed-RILP WT (panel q-t). Cells were fixed and processed for IIF. *C*. *burnetii* was detected with an anti-*C*. *burnetii* antibody // antiserum (panels a-h, red pseudo-colour; panels i-t, white pseudo-colour). Arrows indicate non-transfected cells containing CCV. Scale bar: 10 μm. Quantitative analysis of CCV size (B) and number (C), and bacterial multiplication (D). Forty to sixty cells were analysed in each experiment. Results are expressed as means ± SE of three independent experiments. ***p<0.001. (E) Phase contrast microscopy of infected and transfected HeLa cells. Arrowheads indicate a nrCCV (panels b, d and e), or a CCV (panels a and c). Scale bar: 2 μm.

Infected cells overexpressing hVps41 WT showed a hVps41-positive CCV. Such cells presented 3.0±0.5 vacuoles/cell with a diameter of 7.2±0.5 μm (Fig 6A, panels a-d, 6B and 6C), similarly to control cells (data not shown). Nevertheless, the overexpression of HA-hVps41 A187T induced the formation of smaller (1.0±0.2 μm) and a higher number (22.0 ±1.2 vacuoles/cell) of nrCCVs, as compared to control cells (Fig 6A, panels e-h, 6B and 6C). The multiplication rate of *C*. *burnetii* was inhibited by 71% in cells overexpressing HA-hVps41 A187T, when compared to control cells overexpressing EGFP or cells overexpressing hVps41 WT (Fig 6D). Non-transfected cells presented CCVs (panels e-g, arrow pointed cells). These data suggest that a functional hVps41 is important for the formation of the CCV.

The interplay between Vps41 and RILP is responsible, in part, for the fusion events that take place during the late steps of the endocytic pathway. To study this relationship in during the formation of the CCV, infected cells were co-transfected with plasmids encoding HA-hVps41 WT and DsRed-RILP WT or DsRed-RILP ΔN. The size (6.2±0.2 μm) and the number (2.7±0.2 vacuoles/cell) of CCV formed in cells overexpressing either hVps41 WT and RILP WT (Fig 6A, 6B and 6C) did not differ from that observed in control cells overexpressing EGFP (Fig 6B and 6C). Both proteins were recruited to the CCV membrane (Fig 6A, panels i-l, and Table 1). In contrast, in cells overexpressing the hVps41 WT and RILP ΔN, *C*. *burnetii* resided inside of numerous (23.7±0.2 vacuoles/cell) and small (1.0±0.2 μm) nrCCVs labelled with RILP ΔN (Fig 6A, panels m-p, 6B and 6C), but lacking hVps41 WT. Similar results were observed when RILP WT was co-expressed with hVps41 A187T (3.0±0.2 vacuoles/cell, 6.3±0.2 μm) (Fig 6A, panels q-t, 6B and 6C). Normal CCVs were observed in non-transfected cells (panels m-p and q-t, arrow pointed cells).

The small GTPase Arl8 (Arf-like Small G Protein 8) has been demonstrated to be involved in the HOPS complex recruitment to LE/Ly without affecting the recruitment Rab7 [45]. Apparently, Rab7 is upstream of Arl8. However, it is considered that both GTPases work cooperatively in the recruitment and stabilization of the HOPS complex on endolysosomal membranes [45,78].

**Fig 7:**
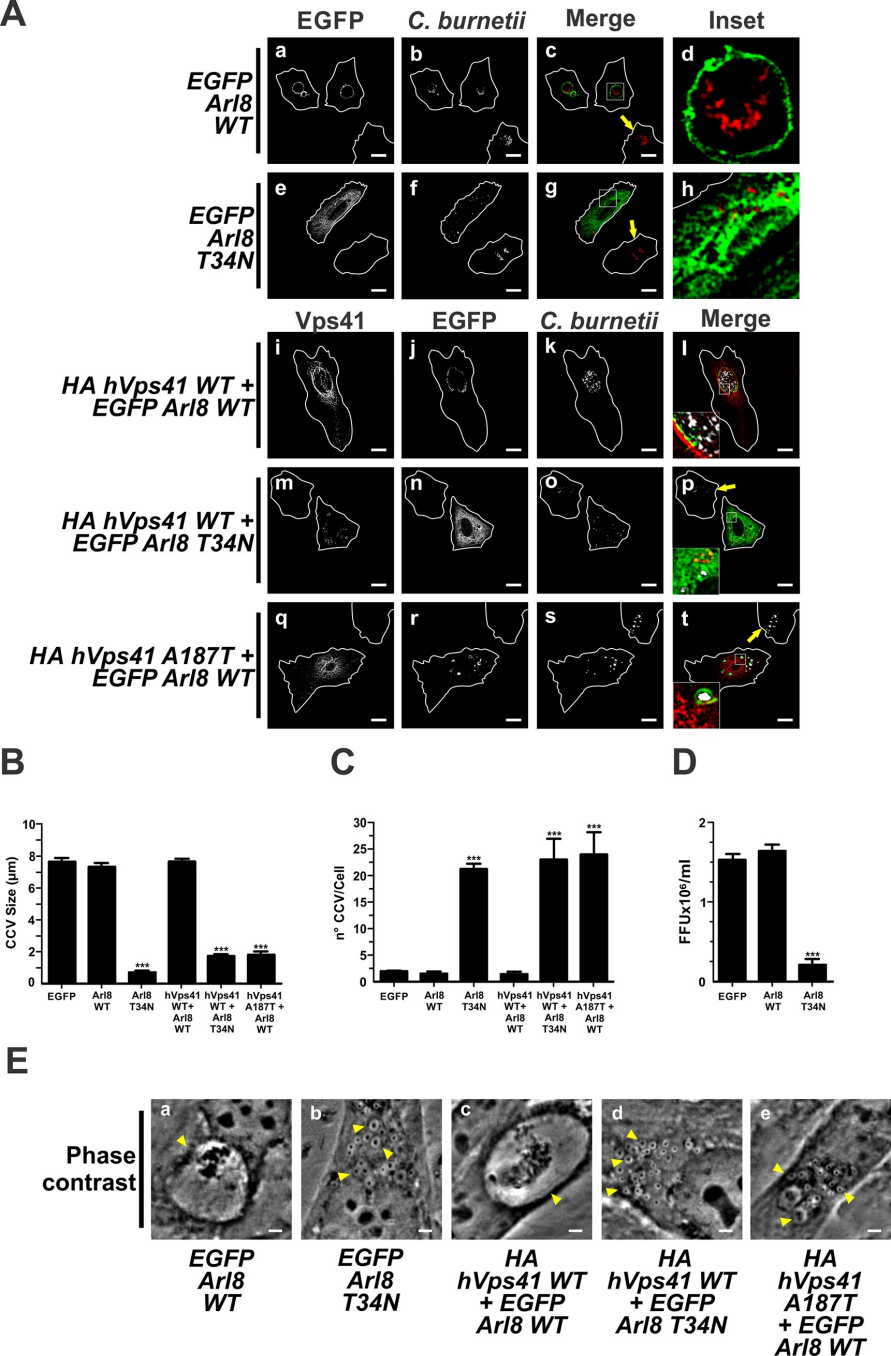
Arl8 and HOPS are involved in the development of the CCV. (A) Infected HeLa cells were transfected with plasmids encoding EGFP-Arl8 WT (panels a-d) or EGFP-Arl8 T34N (panels e-h), or co-transfected with plasmids encoding HA-hVps41 WT and EGFP-Arl8 WT (panels i-l), HA-hVps41 WT and EGFP-Arl8 T34N (panels m-p) or HA-hVps41 A187T and EGFP-Arl8 WT (panels q-t). Cells were fixed and processed for IIF. *C*. *burnetii* and Vps41 were detected with an anti-*C*. *burnetii* antiserum (red pseudo-colour) and an anti-HA (green pseudo-colour) antiserum, respectively. The arrow indicates non-transfected cell containing a CCV. Scale bar: 10 μm. (B) Quantification of (B) size and (C) number of CCV and (D) bacterial multiplication. Forty to sixty cells were analysed in each experiment. Results are expressed as means ± SE of three independent experiments. ***p<0.001. (E) Phase contrast microscopy of infected and transfected HeLa cells. Arrowheads indicate a nrCCV (panels b, d and e), or a CCV (panels a and c). Scale bar: 2 μm.

When cells were infected and then transfected with pEGFP-Arl8 WT, we observed CCVs with a diameter of 7.2±0.5 μm, similarly to that observed in control cells overexpressing EGFP (Fig 7A, panels a-d, and 7B). The number of CCVs (2.0±0.5 vacuoles/cell) was not statistically different from control cells overexpressing EGFP (Fig 7C). Contrarily, the overexpression of EGFP-Arl8 T34N, a constitutively negative mutant [45], induced the formation of smaller (0.5±0.2 μm) and a higher number (23.0±1.2 vacuoles/cell) of nrCCVs (Fig 7A, 7B and 7C) than control cells overexpressing EGFP (Fig 7B and 7C). It was observed that non-transfected cells presented normal CCVs (panels e-h of Fig 7, arrow pointed cells).

The bacterial multiplication was inhibited by 88% in cells overexpressing EGFP-Arl8 T34N compared to that observed in control cells overexpressing EGFP or cells overexpressing EGFP-Arl8 WT (Fig 7D). These results show the importance of this small GTPase in the formation of CCVs.

As demonstrated above, the interaction between the HOPS complex and RILP is important in the formation of the CCV (Fig 6). Arl8 plays a role in the recruitment and stabilization of the HOPS complex on the LE/Ly membrane and in the formation of the CCV. To test if these proteins participate in the formation of the CCV, infected cells were co-transfected with plasmids encoding HA-hVps41 WT and EGFP-Arl8 WT or EGFP-Arl8 T34N. As shown in Fig 7A, panels i-l, 7B and 7C, the size (7.7 ±0.2 μm) and the number (1.5±0.5 vacuoles/cell) of CCVs in cells co-expressing Vps41 WT and Arl8 WT were comparable to those observed in cells overexpressing Arl8 WT (Fig 7A, panels a-d), Vps41 WT (Fig 6A, panels a-d) or EGFP (Fig 7B and 7C). Co-expressed Vps41 WT and Arl8 WT were found to be recruited to CCVs (Fig 7A, panels i-l, and Table 1). In contrast, in cells overexpressing both Arl8 T34N and Vps41 WT, *C*. *burnetii* resided inside of numerous (22.5±5 vacuoles/cell) and small (1.7±0.2 μm) nrCCVs labelled with Arl8 T34N (Fig 7A, panels m-p, 7B and 7C, and Table 1) but negative for hVps41 WT. Regarding the size and number of CCVs, similar results were observed when Arl8 WT was co-expressed with hVps41 A187T (Fig 7A, panels q-t, 7B and 7C). Arl8 WT, but not hVps41 A187T, was recruited to nrCCVs (Fig 7A, panels q-t, and Table 1). In conclusion, these results show that Vps41 is recruited to the CCV but only in the presence of the active forms of Arl8 and RILP.

## Discussion

In this report, we show that Mts and the Mts-associated motors dynein and kinesin play very important roles in the biogenesis of the *C*. *burnetii*-containing vacuoles (CCV) and the intracellular bacterium multiplication. This is the first molecular description of the interplay between the CCV and Mts-motor proteins. Mts and motor proteins are used by several bacteria to accomplish cell invasion, intracellular trafficking and intra- and inter-cellular spreading [11,79,80–87,88].

Herein we demonstrate that the biogenesis of the CCV is a Mts and motor proteins-dependent process (Figs 1 and 3). The results showing the inhibitory effect of both nocodazole and taxol on the formation of CCV and bacterial replication suggest that Mts should be dynamic.

**Fig 8:**
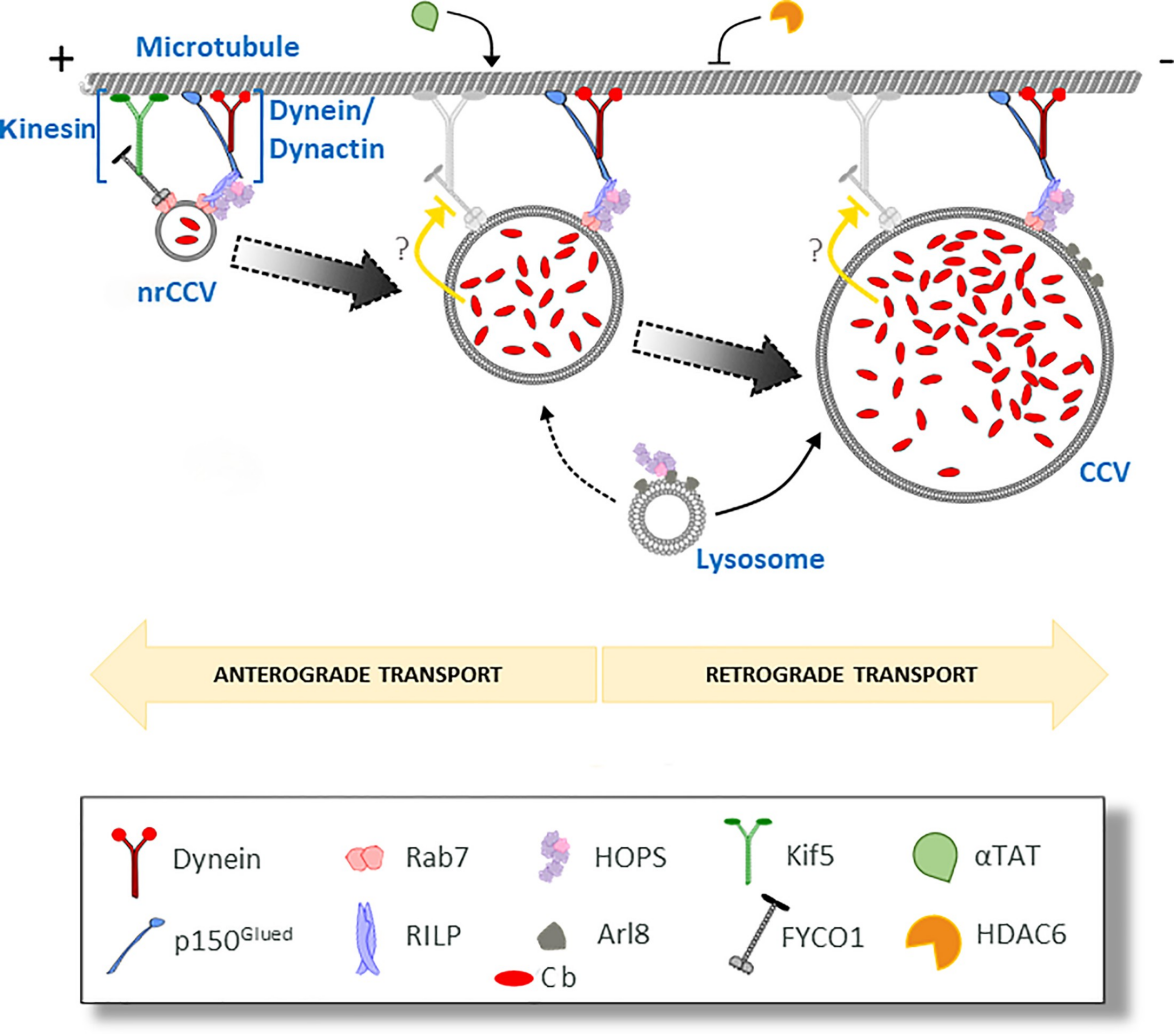
Relationship of intracellular transport of *C*. *burnetii* with microtubules and motor proteins: A model. *C. burnetii* (Cb) transits along the endo-phagocytic pathway into non-replicative *C. burnetii*-containing vacuole (nrCCVs) acquiring markers such as Rab7. This small GTPase recruits RILP protein, dynein/dynactin motor and HOPS complexes to the nrCCVs. This molecular machinery drives a gradual retrograde transport along Mts and the fusion of the nrCCVs with each other and with other endocytic compartments such as lysosomes. The fusion of lysosomes with nrCCVs is stimulated by the small GTPase Arl8 and HOPS complex. To the end of this journey, the *C. burnetii*-containing vacuole (CCV) is formed. Dynamic Mts and their acetylation-deacetylation status, regulated by acetyl transferase and deacetylase, are also important for CCV formation. *C. burnetii* could inhibit kinesin/FYCO1 (orange arrows) favouring the retrograde transport driven by the dynein/dynactin motor complex. This condition leads to the formation of the CCV. The following mechanism can explain the inhibition of the formation of the CCV by the expression of kinesin or FYCO1: the balance between dynein and kinesin recruited to nrCCVs can be shifted in favour of kinesin therefore the nrCCVs acquire a Mts-mediated anterograde movement that disperses them in the cytoplasm (not shown in the model).

Further evidence regarding the involvement of Mts in the formation of the CCV comes from post-translational modifications studies of tubulin, such as acetylation. We consider that HDAC6 and αTAT are important for the development of the CCV (Fig 2 and 8). The formation of CCVs is favoured when Mts are acetylated, i.e. when the HDAC6 mutant and αTAT are overexpressed WT; however, we cannot asseverate that Mts acetylation is the only factor affecting the formation of the CCV formation.

The acetylation of α-tubulin and αTAT decreases stability and an increases the dynamics of Mts [23,24]; however, opposite results have been reported when working with HDAC6 [58–60].

The relationship between tubulin acetylation and microtubule stability remains controversial; however, our results with nocodazole (a Mts depolymerising agent) and taxol (a Mts stabilizing agent) suggest that Mts should display a dynamic behavior to support CCV biogenesis.

Several authors have demonstrated that the post-translational modification of tubulin, together with the dynamics of Mts, are essential not only for the interaction between Mts and the motor proteins but also for the regulatory functions associated with Mts [18,27,54,67,68]. Although there is not a consensus about the role of acetylation in the affinity of motor proteins for Mts, we have observed that the recruitment of dynactin to the CCV is stimulated by mutated SIRT2 (S2 and S4 Figs, and Table 1) promoting the formation of the CCV. This observation suggests that acetylation could be important in such processes. Accordingly, Gao *et al*. have shown that the binding of dynein motor to acetylated Mts is stimulated by the inhibition and knockdown of HDAC6, which increased the retrograde transport of endosomes containing EGFR toward the late degradative endosomal compartment [27]. These findings support our hypothesis suggesting that the recruitment of dynein motor drives nrCCV retrograde trafficking and, therefore, the formation of the CCV (Fig 8).

The formation of the CCV also depends on Mts-associated motor proteins. The overexpressed p150^Glued^WT, a dynactin subunit, is recruited to the CCV in a Rab7/RILP-dependent fashion; in addition, it is also important for the biogenesis of the CCV, since its non-functional mutant p150^Glued^CC1 inhibits the formation of the CCV (Fig 3). This finding is in line with the formation of numerous small nrCCVs in cells overexpressing p50^dynamitin^. It is known that the overexpression of p50^dynamitin^ disrupts the dynactin complex and the dynein motor function. These results suggest that the dynactin complex plays an important role in the CCV biogenesis (Fig 8). Interestingly, other intracellular pathogens use the same molecular machinery to develop their replicative niches. For instance, the initial intracellular transport of *Salmonella*-containing vacuoles (SCV) to and its maintenance in the juxtanuclear region require the dynein-dynactin complex [89]. A dispersion of bacteria was observed when either RILP C33 or p50^dynamitin^ were overexpressed [90]. Similar results have been observed for inclusions containing *Chlamydia trachomatis* in infected cells [87].

Rab7 mediates the recruitment of dynein to late endosomes through its effector RILP [36,70]. Herein, we demonstrate that RILP is also recruited to the CCV in a Rab7-dependent manner. In addition, RILP is required for dynein recruitment to the CCV (Fig 4, and Table 1), since this association was prevented by the overexpression of the RILP ΔN, a truncated form of RILP. Under these conditions, the CCV is not observed.

Cantalupo *et al*. have suggested the existence of a direct interaction between Rab7 and RILP [70]. The results obtained in that work are in line with the results presented herein. Jordens *et al*. have proposed RILP as a motor complex adapter protein [36]. We demonstrated that the dynein/dynactin motor complex is not only associated with the CCV but also that this complex must be functional to accomplish the formation of the CCV. Our results would suggest that the fusion of nrCCVs to generate the CCV was favoured by the motor complex and RILP (Fig 4).

In epithelial cells, the formation of *Salmonella*-induced filaments (SIFs) depends on the integrity and transport function of Mts [91–93]. SIF membranes recruit kinesin instead of dynein-RILP [89,90,94].

PipB2 (a SPI2-T3SS *Salmonella* effector) and Arl8 (a host ARF GTPase) stimulate the kinesin-1 recruitment to the SCV [95–97]. This activity is counterbalanced by the interaction of SifA (SifA-kinesin interacting protein) with SKIP (SifA-kinesin interacting protein), leading to the partial exclusion of kinesin-1 from the SCV and the proper positioning of the SCV. In the absence of SifA or SKIP, the SCV associated with kinesin, thus leading to further Mts-dependent anterograde transport and, ultimately, to the breaking of the SCV [97–99]. In our system, in infected cells overexpressing KIF5 WT (kinesin-1) or FYCO WT, several nrCCVs were observed instead of the CCV (Fig 5). It could be speculated that the CCV disrupts into small vacuoles, as it occurs in *Salmonella* infected cells. This hypothesis seems to be unlikely since the CCV was not disrupted when the cells were transfected with the plasmid encoding KIF5B after the formation of the CCV (48h post-infection, unpublished data). Other possibility is that the kinesin-mediated anterograde transport of nrCCVs is stimulated, thus hampering their retrograde movement and preventing the homotypic fusion to form the CCV (Fig 8). Our results suggest that *C*. *burnetii* would inhibit both the binding of kinesin to the CCV and the anterograde transport, thus stimulating the retrograde one (Fig 8). These hypotheses are under current study in our laboratory. It is known that the HOPS complex regulates the late stage of the endocytic pathway, driving late endosomal membrane tethering and fusion. Vps41 functions as a nexus between HOPS and RILP [41] and this interaction allows R- and Q-SNAREs association and membrane fusion [100]. The Rab7-RILP association also brings together the HOPS and the dynein motor complexes for retrograde transport [101]. It has been demonstrated that Vps41 co-localises with avirulent *C burnetii* in a p38a-MAPK-dependent manner [102]. Our results demonstrate that both the Vps41 and RILP associated to and are required for the formation of the CCV (Fig 6 and Table 1). In cells overexpressing RILP and/or Vps41 non-functional mutants we observed nrCCVs instead of CCV detected in cells overexpressing WT RILP and/or WT Vps41 (Fig 6). We hypothesise that the recruitment of HOPS complex and RILP would stimulate the homotypic fusion among nrCCVs and with different compartments, thus promoting the formation of the CCV (Fig 8).

Not only does Rab7-RILP interact with HOPS to regulate membrane traffic toward lysosomes, but also the Arl8 GTPase [103]. Garg *et al*. [104] have demonstrated the importance of Arl8 in antigen presentation and pathogen killing by regulating phagolysosome fusion. In this report, we show that the overexpressed Arl8 WT localizes to the CCV, while the overexpression of its mutant Arl8 T34A produces nrCCVs that are negative for this protein (Fig 7 and Table 1). Therefore, Arl8 is required for the formation of the CCV. It is known that Vps41 is an effector of Arl8 and the interaction between them occurs in lysosomes which fuse with late endosomes decorated with Rab7 [43]. The co-localization of Rab7, Vps41 and Arl8 in the CCV would suggest that the CCV is generated by fusion of nrCCVs and/or CCV with lysosomes (Table 1).

Arl8 interacts with SKIP (Sif-A and kinesin-interacting protein) which binds kinesin-1 to mediate the anterograde lysosomal movement [35,45]. The recruitment of dynein and kinesin motors to SCV is important for *Salmonella* survival. This has been demonstrated by Mrakovic *et al*. [35], who have shown that the tubulation of lysosomes (an important effect for *Salmonella* survival within the host cell) is orchestrated by dynein and kinesin recruited to the SCV by Rab7 and Arl8, respectively.

In our model, the expression of Arl8 alone allowed the formation of the CCV, while the expression of KIF5B alone stimulated the formation of several nrCCVs with small sizes, as compared to the CCV. We believe that the overexpression of KIF5B shifts the kinesin-dynein equilibrium towards kinesin stimulating anterograde transport and dispersion of small nrCCVs that can neither aggregate nor fuse with each other or with lysosomes to form the CCV (Fig 8). On the contrary, the Arl8-positive CCV forms in cells overexpressing Arl8 alone. We believe that under these conditions, Arl8 interacts with the endogenous downstream effectors stimulating the fusion of CCV and/or nrCCVs with lysosomes. As mentioned above, some of these hypotheses remain to be tested.

In conclusion, in the present report we demonstrate that dynamic Mts and enzymes involved in acetylation-deacetylation of α-tubulin play important roles in the biogenesis of the CCV and intracellular bacterial multiplication. Furthermore, we prove that RILP and its partner Rab7 are involved in the recruitment of dynein/dynactin motor and HOPs complexes to nrCCVs and CCV. Considering these multiple interactions, we propose that the dynein complex stimulates the Mts-dependent retrograde trafficking of nrCCVs, and that HOPs allows tethering and homotypic and heterotypic fusion events that ultimately lead to the CCV formation. In addition, we present results suggesting that the GTPase Arl8 would contribute to CCV development by stimulating anterograde transport and fusion of lysosomes with nrCCVs and/or with CCV (Fig 8).

## Supporting information

S1 Fig Effect of nocodazol and taxol upon HeLa cell viability. HeLa cells were seeded in 24-well plates and grown overnight. Then, cells were incubated at 37°C for different periods of time with DMSO (0.1%), nocodazol (2μM, Noc) or taxol (2μM). Culture media were transferred to 15 ml tubes (non-attached cells) and kept on ice; and the attached flattened cells were trypsinised. After washing twice, these cells were transferred to 15 ml tubes containing unattached cells. Tubes were centrifuged at 200 xg for 5 min at 4°C. Cell pellets were resuspended in PBS and processed to estimated cell viability by using the Trypan blue exclusion test according to standard protocols. Cell viability is expressed as percentage of the total cells relative to control cells (DMSO). Data represent the mean ± SE of three independent experiments. p < 0.05.
(TIF)

S2 Fig The overexpression of the deacetylase SIRT2 inhibits the formation of CCV. Infected HeLa cells were transfected with plasmids encoding HA-SIRT2 WT (panels a-d) or HA-SIRT2 NLSΔNES (panels e-h). Cells were fixed and processed for IIF. *C*. *burnetii* and SIRT2 were detected with an anti-*C*. *burnetii* antiserum (red pseudo-colour) and an anti-HA antiserum (green pseudo-colour), respectively. Scale bar: 10 μm. Quantitative analysis of CCV size (B) and number (C), and bacterial multiplication (D). Forty to sixty cells were analysed in each experiment. Results are expressed as means ± SE of three independent experiments. ***p< 0.001. (E) Phase contrast microscopy of infected and transfected HeLa cells. Arrowheads indicate a nrCCV (panel a), or a CCV (panel b). Scale bar: 2 μm.
(TIF)

S3 Fig Detection of acetylated microtubules in infected cells overexpressing EGFP-HDAC6 or -αTAT. Infected HeLa cells were transfected with pEGFP-HDAC6WT (panels a-d) or -αTAT WT (panels e-h). Cells were fixed and processed for IIF. Anti-*C. burnetii* and anti-acetylated α-tubulin antisera (Sigma-Aldrich, Argentina) were used for detecting bacteria (grey pseudo-colour, panels c and g) and acetylated microtubules (red pseudo-colour, panels b and f), respectively. Arrows indicate non-transfected cells containing a CCV. Scale bar: 10 μm. (B) Phase contrast microscopy of infected and transfected HeLa cells. Arrowheads indicate a nrCCV (panel a), or a CCV (panel b). Scale bar: 2 μm.
(TIF)

S4 Fig The overexpression of the deacetylase SIRT2 inhibits 150^Glued^WT recruitment and the formation of the CCV. (A) Infected HeLa cells were co-transfected with plasmids encoding EGFP-p150^Glued^WT and HA-SIRT2 WT (panels a-d) or EGFP-p150^Glued^WT and HA-SIRT2 NLSΔNES (panels e-h). Cells were fixed and processed for IIF. *C*. *burnetii* and HA-SIRT2 were detected with anti-*C*. *burnetii* (green pseudo-colour) and anti-HA (red pseudo-colour) antisera, respectively. Yellow arrows indicate non-transfected cell containing CCV. Scale bar: 10 μm. Quantitative analysis of CCV size (B) and number (C). Forty to sixty cells were analysed in each experiment. Results are expressed as means ± SE of three independent experiments. ***p<0.001. (D) Phase contrast microscopy of infected and transfected HeLa cells. Arrowheads indicate a nrCCV (panel a), or a CCV (panel b). Scale bar: 2 μm.
(TIF)

S5 Fig RILP is required for the formation of the *C*. *burnetii*-containing vacuole. Infected HeLa cells were co-transfected with pEGFP-empty vector (A) or pEGFP-RILP WT (B) with scramble-siRNA (panels a-b), RILP-siRNA 1 (panels c-d) or RILP-siRNA 2 (panels e-f) (siRNAs purchased from Bioneer, Inc. Alameda, USA). Cells were fixed and processed for IIF using an anti-*C*. *burnetii* antiserum (red pseudo-colour). Scale bar: 5 μm. Quantitative analysis of CCV size (C) and number (D). Forty to sixty cells were analysed in each experiment. Results are expressed as means ± SE of three independent experiments. ***p<0.001. (E) HeLa cells were co-transfected with pEGFP-RILP WT and scramble-siRNA (line 1), RILP-siRNA 1 (line 2) or RILP-siRNA 2 (line 3). Cell lysate proteins were separated by SDS-PAGE and analysed by Western blotting using antibodies against GFP (Genscript USA Inc., USA) or tubulin (loading control) (Sigma-Aldrich Inc., Argentina). (F) HeLa cells were transfected with scramble-siRNA (line 1), RILP-siRNA 1 (line 2) or RILP-siRNA 2 (line 3). Cell lysate proteins were separated by SDS-PAGE and analysed by Western blotting using antibodies against RILP (Santa Cruz Biotechnology Inc., USA) or tubulin (loading control). Molecular weight standards are indicated with arrowheads. (G) Bands corresponding to overexpressed EGFP-RILP WT and endogenous RILP were quantified (relative to tubulin) using the ImageJ software. Results are expressed as means ± SD of two independent experiments. ***p<0.05.
(TIF)

S6 Fig The formation of CCV in cells expressing RILP is inhibited by the expression of the dominant negative mutant Rab7 T22N. (A) Infected HeLa cells were co-transfected with plasmids encoding pDsRed-RILP WT and pEGFP-Rab7 T22N (panels a-d) or pDsRed-RILP WT and pEGFP-Rab7 Q67L (panels e-h). Cells were fixed and processed for IIF. *C*. *burnetii* was detected with an anti-*C*. *burnetii* antiserum (white pseudo-colour). Scale bar: 10 μm. Quantitative analysis of CCV size (B) and number (C). Forty to sixty cells were analysed in each experiment. Results are expressed as means ± SE of three independent experiments. ***p<0.001. (D). Phase contrast microscopy of infected and transfected HeLa cells. Arrowheads indicate a nrCCV (panel a), or a CCV (panel b). Scale bar: 2 μm.
(TIF)

S1 Table Experimental CCV mesures. Infected HeLa cells were either transfected or co-transfected with plasmids encoding the different proteins under study. An average of 50 cells per coverslip was calculated (in triplicate) to determine the diameter and number of vacuoles containing *C*. *burnetii*. Images were acquired with a Nikon Eclipse TE2000 microscope and analysed by phase contrast microscopy and assumptions with the fluorescence image to be able to observe the CCV correctly. The size and number of CCV were calculated by means of a morphometric analysis using the different measurement tools of the ImageJ software.
(PDF)
