# Supplementary figures and images for: The role of microtubules and the dynein/dynactin motor complex of host cells in the biogenesis of the Coxiella burnetii-containing vacuole
Source: PLoS One. 2019 Jan 14;14(1):e0209820. doi: 10.1371/journal.pone.0209820 (PMC6331085; doi:10.1371/journal.pone.0209820)

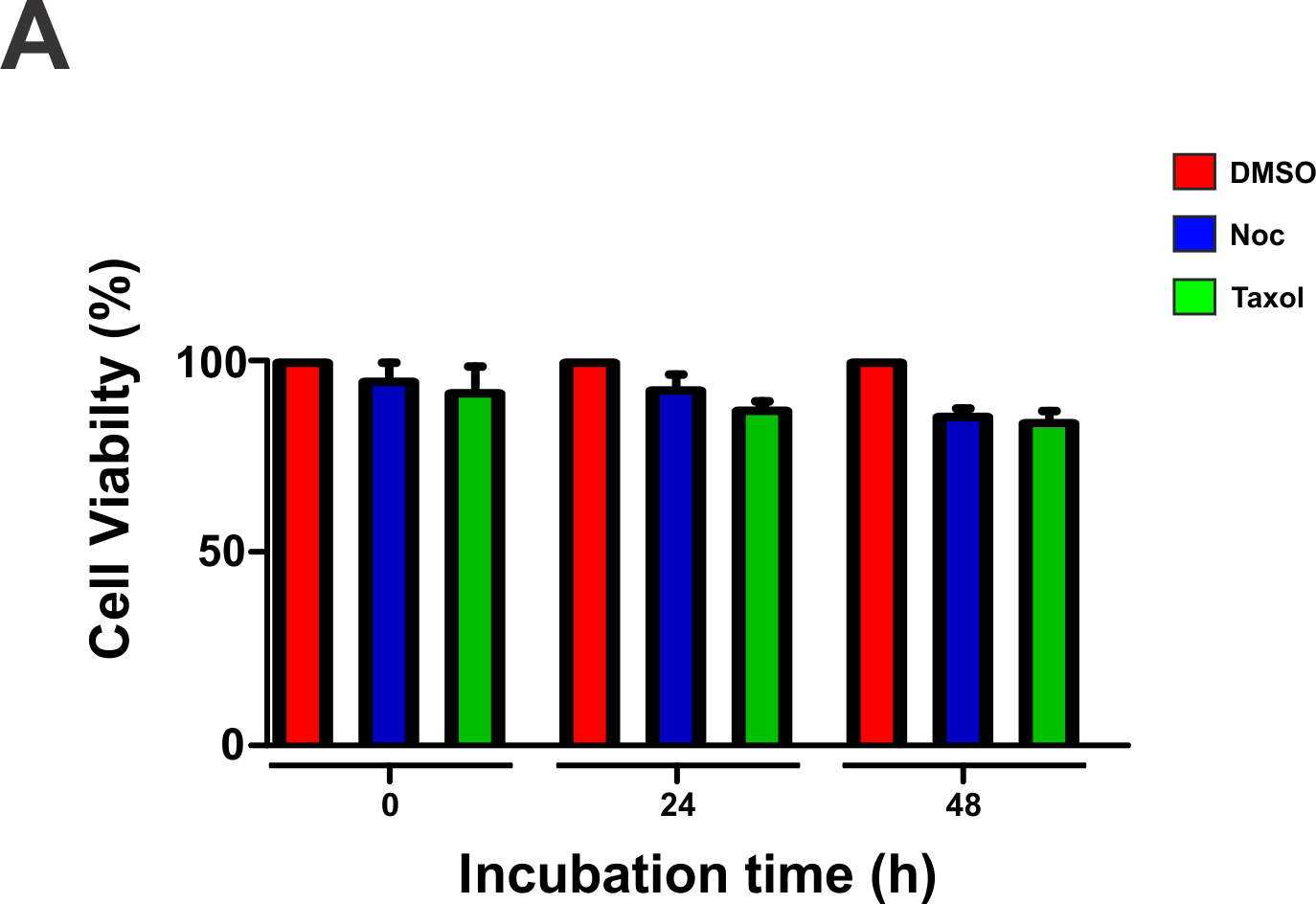

Supplement: S1 Fig — HeLa cells were seeded in 24-well plates and grown overnight. Then, cells were incubated at 37°C for different periods of time with DMSO (0.1%), nocodazol (2μM, Noc) or taxol (2μM). Culture media were transferred to 15 ml tubes (non-attached cells) and kept on ice; and the attached flattened cells were trypsinised. After washing twice, these cells were transferred to 15 ml tubes containing unattached cells. Tubes were centrifuged at 200 xg for 5 min at 4°C. Cell pellets were resuspended in PBS and processed to estimated cell viability by using the Trypan blue exclusion test according to standard protocols. Cell viability is expressed as percentage of the total cells relative to control cells (DMSO). Data represent the mean ± SE of three independent experiments. p < 0.05. (TIF) [file pone.0209820.s001.tif]

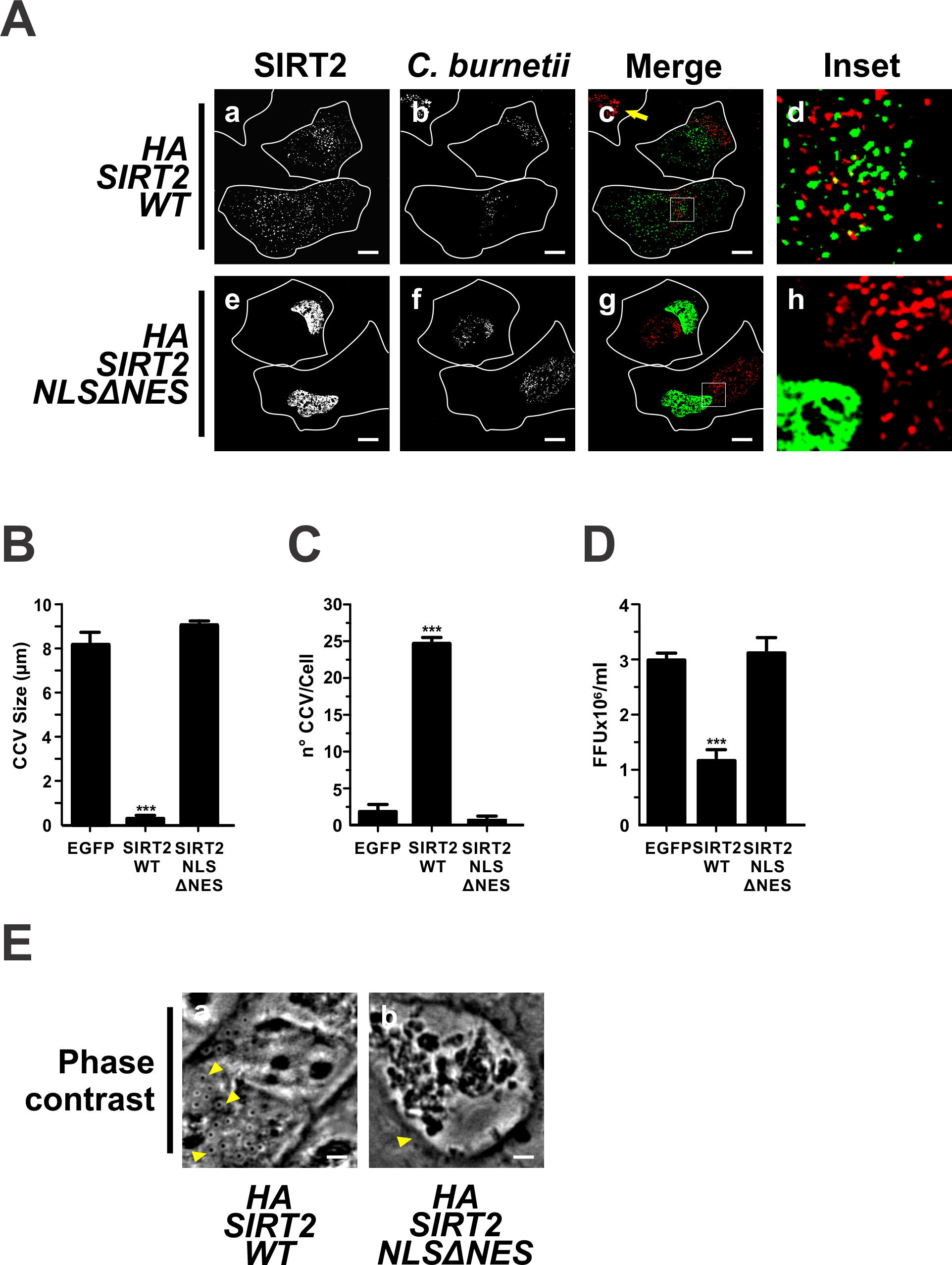

Supplement: S2 Fig — Infected HeLa cells were transfected with plasmids encoding HA-SIRT2 WT (panels a-d) or HA-SIRT2 NLSΔNES (panels e-h). Cells were fixed and processed for IIF. C. burnetii and SIRT2 were detected with an anti-C. burnetii antiserum (red pseudo-colour) and an anti-HA antiserum (green pseudo-colour), respectively. Scale bar: 10 μm. Quantitative analysis of CCV size (B) and number (C), and bacterial multiplication (D). Forty to sixty cells were analysed in each experiment. Results are expressed as means ± SE of three independent experiments. ***p< 0.001. (E) Phase contrast microscopy of infected and transfected HeLa cells. Arrowheads indicate a nrCCV (panel a), or a CCV (panel b). Scale bar: 2 μm. (TIF) [file pone.0209820.s002.tif]

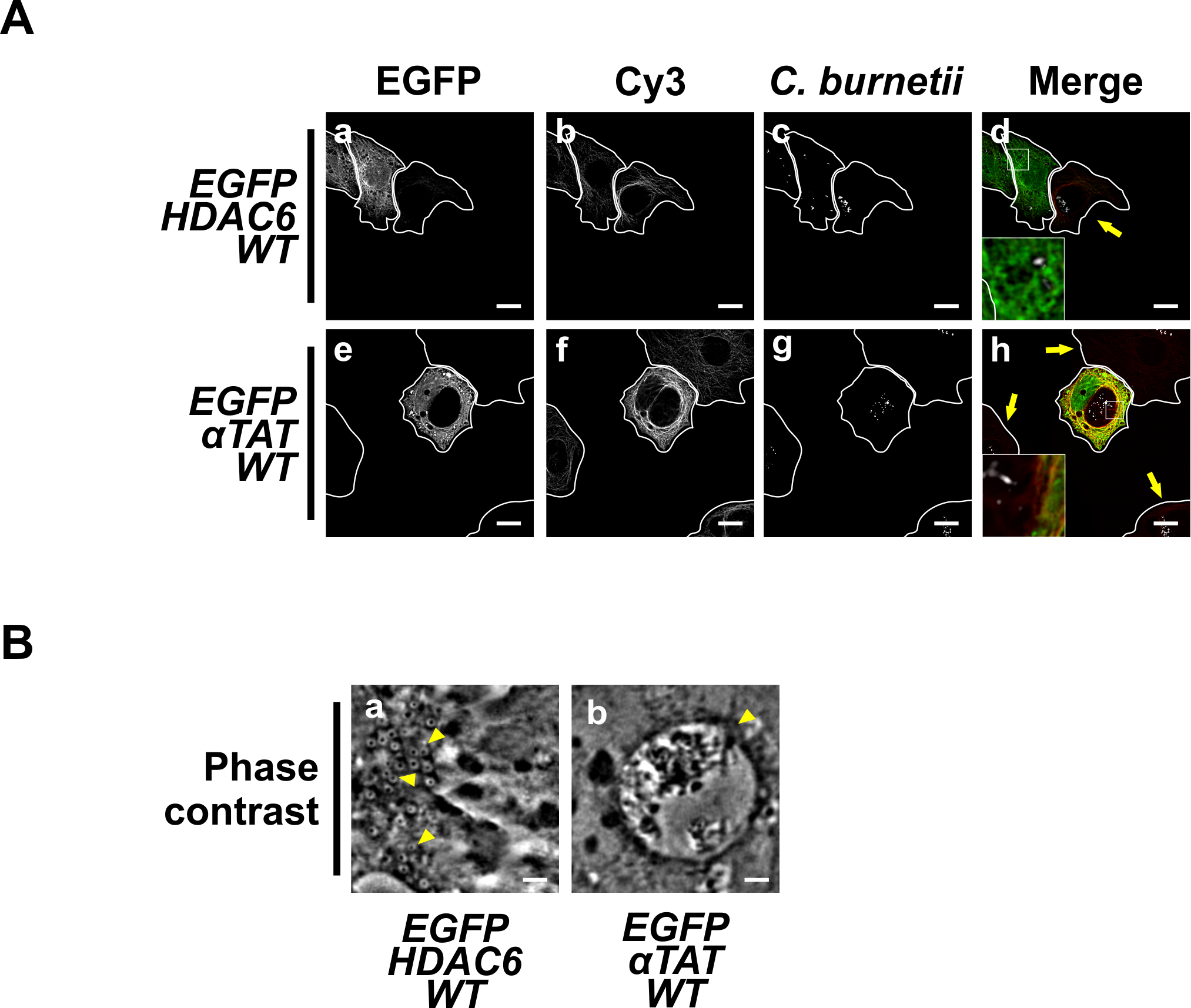

Supplement: S3 Fig — Infected HeLa cells were transfected with pEGFP-HDAC6WT (panels a-d) or -αTAT WT (panels e-h). Cells were fixed and processed for IIF. Anti-C. burnetii and anti-acetylated α-tubulin antisera (Sigma-Aldrich, Argentina) were used for detecting bacteria (grey pseudo-colour, panels c and g) and acetylated microtubules (red pseudo-colour, panels b and f), respectively. Arrows indicate non-transfected cells containing a CCV. Scale bar: 10 μm. (B) Phase contrast microscopy of infected and transfected HeLa cells. Arrowheads indicate a nrCCV (panel a), or a CCV (panel b). Scale bar: 2 μm. (TIF) [file pone.0209820.s003.tif]

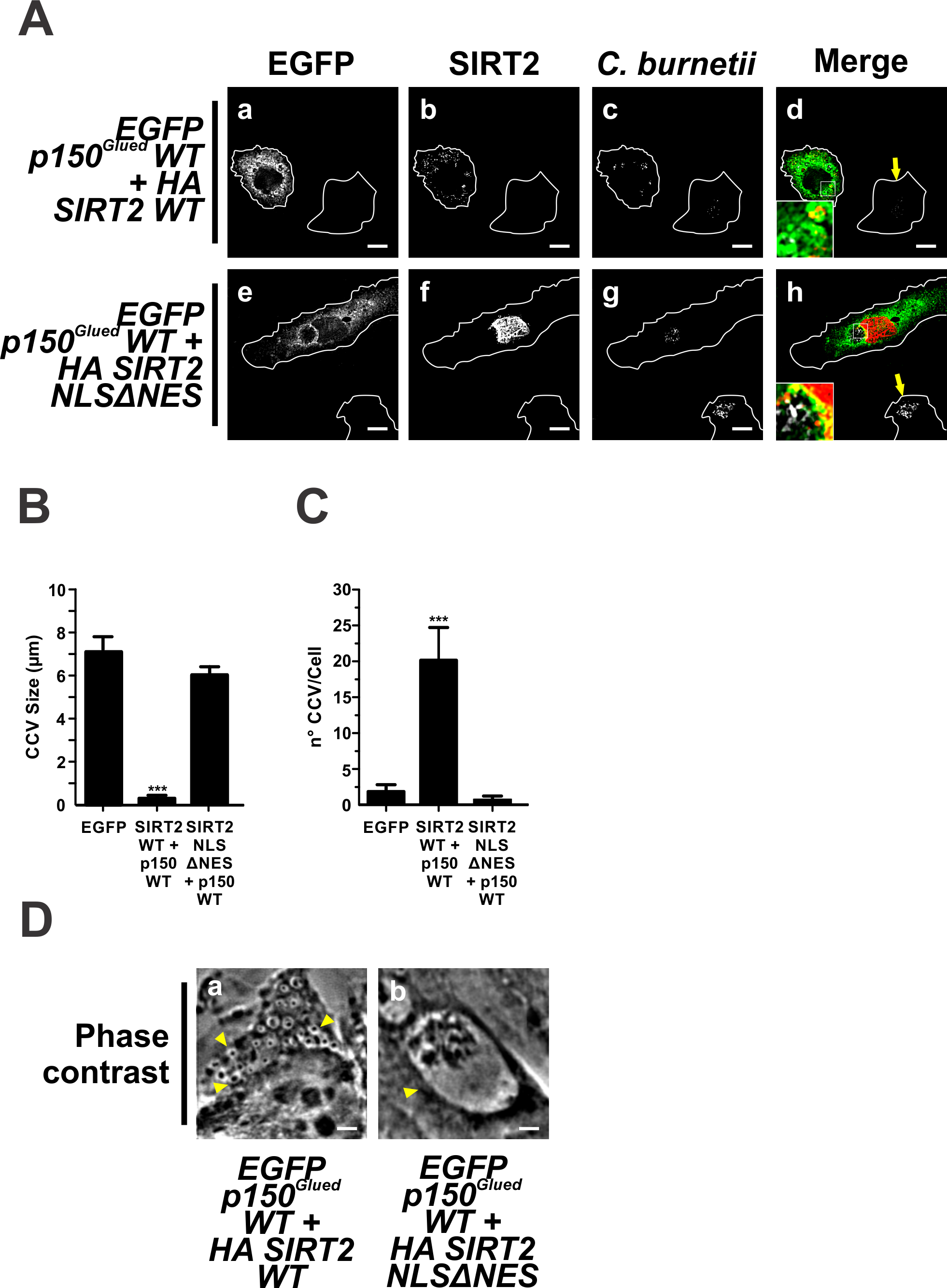

Supplement: S4 Fig — (A) Infected HeLa cells were co-transfected with plasmids encoding EGFP-p150GluedWT and HA-SIRT2 WT (panels a-d) or EGFP-p150GluedWT and HA-SIRT2 NLSΔNES (panels e-h). Cells were fixed and processed for IIF. C. burnetii and HA-SIRT2 were detected with anti-C. burnetii (green pseudo-colour) and anti-HA (red pseudo-colour) antisera, respectively. Yellow arrows indicate non-transfected cell containing CCV. Scale bar: 10 μm. Quantitative analysis of CCV size (B) and number (C). Forty to sixty cells were analysed in each experiment. Results are expressed as means ± SE of three independent experiments. ***p<0.001. (D) Phase contrast microscopy of infected and transfected HeLa cells. Arrowheads indicate a nrCCV (panel a), or a CCV (panel b). Scale bar: 2 μm. (TIF) [file pone.0209820.s004.tif]

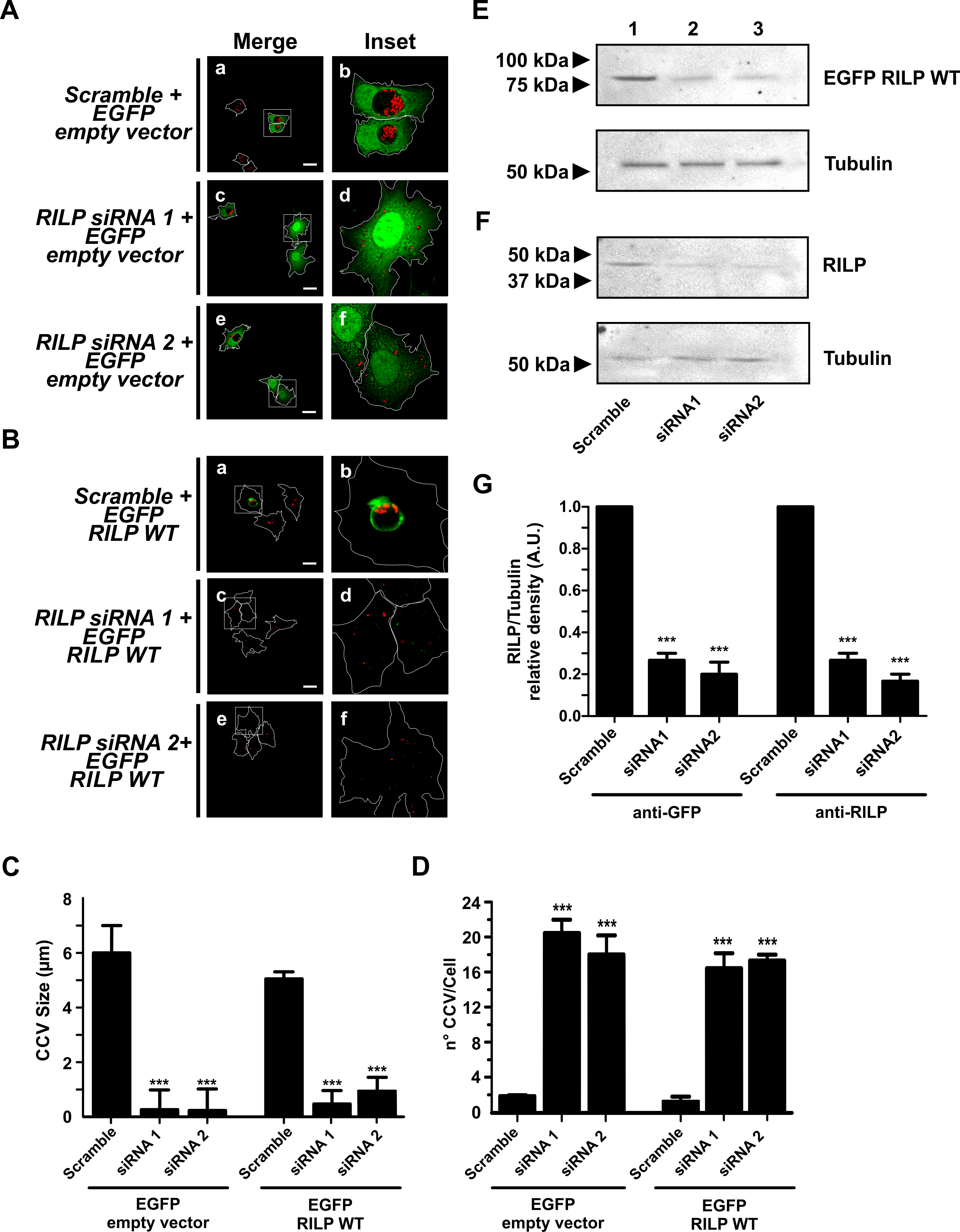

Supplement: S5 Fig — Infected HeLa cells were co-transfected with pEGFP-empty vector (A) or pEGFP-RILP WT (B) with scramble-siRNA (panels a-b), RILP-siRNA 1 (panels c-d) or RILP-siRNA 2 (panels e-f) (siRNAs purchased from Bioneer, Inc. Alameda, USA). Cells were fixed and processed for IIF using an anti-C. burnetii antiserum (red pseudo-colour). Scale bar: 5 μm. Quantitative analysis of CCV size (C) and number (D). Forty to sixty cells were analysed in each experiment. Results are expressed as means ± SE of three independent experiments. ***p<0.001. (E) HeLa cells were co-transfected with pEGFP-RILP WT and scramble-siRNA (line 1), RILP-siRNA 1 (line 2) or RILP-siRNA 2 (line 3). Cell lysate proteins were separated by SDS-PAGE and analysed by Western blotting using antibodies against GFP (Genscript USA Inc., USA) or tubulin (loading control) (Sigma-Aldrich Inc., Argentina). (F) HeLa cells were transfected with scramble-siRNA (line 1), RILP-siRNA 1 (line 2) or RILP-siRNA 2 (line 3). Cell lysate proteins were separated by SDS-PAGE and analysed by Western blotting using antibodies against RILP (Santa Cruz Biotechnology Inc., USA) or tubulin (loading control). Molecular weight standards are indicated with arrowheads. (G) Bands corresponding to overexpressed EGFP-RILP WT and endogenous RILP were quantified (relative to tubulin) using the ImageJ software. Results are expressed as means ± SD of two independent experiments. ***p<0.05. (TIF) [file pone.0209820.s005.tif]

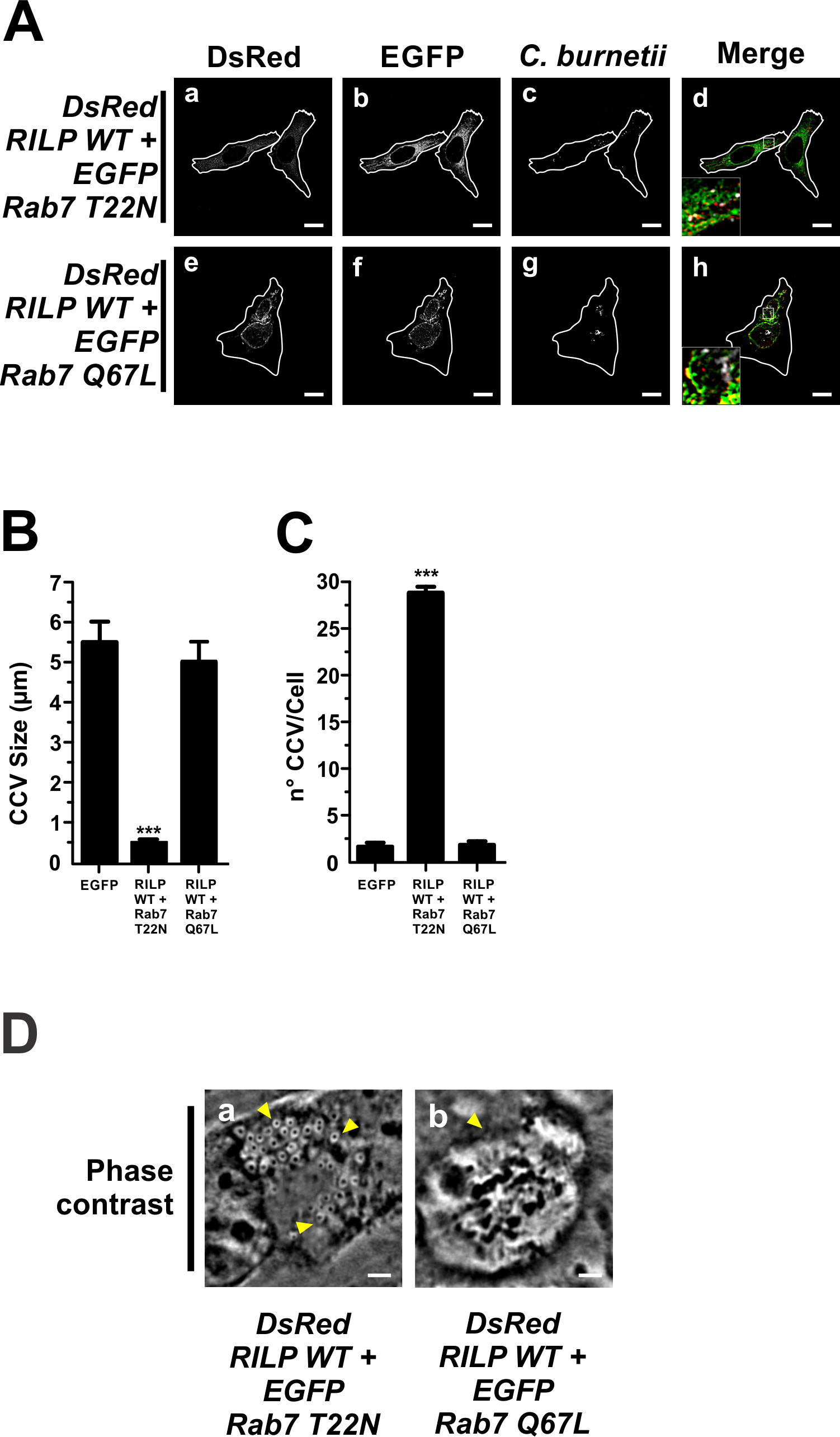

Supplement: S6 Fig — (A) Infected HeLa cells were co-transfected with plasmids encoding pDsRed-RILP WT and pEGFP-Rab7 T22N (panels a-d) or pDsRed-RILP WT and pEGFP-Rab7 Q67L (panels e-h). Cells were fixed and processed for IIF. C. burnetii was detected with an anti-C. burnetii antiserum (white pseudo-colour). Scale bar: 10 μm. Quantitative analysis of CCV size (B) and number (C). Forty to sixty cells were analysed in each experiment. Results are expressed as means ± SE of three independent experiments. ***p<0.001. (D). Phase contrast microscopy of infected and transfected HeLa cells. Arrowheads indicate a nrCCV (panel a), or a CCV (panel b). Scale bar: 2 μm. (TIF) [file pone.0209820.s006.tif]
