# Supplementary material for: The role of microtubules and the dynein/dynactin motor complex of host cells in the biogenesis of the Coxiella burnetii-containing vacuole
Source: PLoS One. 2019 Jan 14;14(1):e0209820. doi: 10.1371/journal.pone.0209820 (PMC6331085; doi:10.1371/journal.pone.0209820)
Supplement: S1 Table — Infected HeLa cells were either transfected or co-transfected with plasmids encoding the different proteins under study. An average of 50 cells per coverslip was calculated (in triplicate) to determine the diameter and number of vacuoles containing C. burnetii. Images were acquired with a Nikon Eclipse TE2000 microscope and analysed by phase contrast microscopy and assumptions with the fluorescence image to be able to observe the CCV correctly. The size and number of CCV were calculated by means of a morphometric analysis using the different measurement tools of the ImageJ software. (PDF) [file pone.0209820.s007.pdf]

S1 Table. Experimental CCV mesures.

CCV measures					
Figure 1					
CCV size (nm)			n° CCV/Cell		
DMSO	Taxol	Nocodazol	DMSO	Taxol	Nocodazol
10074	2578	934	1	10	14
5113	2521	897	1	11	14
5410	2520	809	1	11	14
5811	3337	905	1	12	14
2791	1245	1033	2	12	14
4976	1805	1040	2	12	14
4023	3680	682	1	10	14
4800	1474	1272	2	10	14
3599	1463	973	1	10	14
5384	2207	1038	2	10	14
4459	4578	907	2	10	14
5520	1288	794	3	10	14
8574	1564	801	3	10	14
4587	4300	594	2	10	14
3866	1196	1099	1	10	14
3726	1121	811	2	10	14
3983	1459	1109	3	10	14
5224	1266	914	2	10	9
3318	3337	921	1	10	9
4655	1805	1362	2	10	10
5683	1520	1280	3	10	12
3928	3245	840	2	10	12
2484	1207	1349	1	10	13
5527	2967	734	2	10	14
3036	1969	842	3	10	14
5437	2145	1636	2	10	12
5325	1518	1007	2	10	12
4287	1380	1026	2	10	13

5895	1568	1050	1	10	15
6111	6578	953	2	10	12
3729	1474	1022	1	10	10
3280	7954	891	1	10	11
3174	3219	895	1	10	11
5260	3795	1157	1	10	11
3039	1347	1109	1	10	14
3315	2208	826	1	10	12
4140	2208	938	1	10	12
7315	1584	934	1	10	13
7621	1983	839	1	10	14
6962	1452	1319	2	10	12
3599	1138	753	2	10	12
6630	834	1231	2	10	13
3729	1230	1230	2	10	15
4694	2277	1163	1	10	12
3643	3070	899	2	10	10
3844	3601	1058	2	10	11
3030	2234	996	2	10	14
5811	975	996	2	10	12
3312	1244	1036	2	8	12
3682	1632	967	2	12	13
6661	2342	725	2	12	15
3365	1168	760	1	12	12
1225	1583	519	1	12	10
4258	1172	518	1	12	11
2658	1923	656	1	12	11
3254	1255	622	1	12	11
2648	1391	1070	1	12	11
3232	3485	1075	2	12	11
6594	1311	1069	2	12	11
5784	1214	794	1	8	11
4458	2943	794	2	8	14
6648	2741	932	2	8	13

1235	1130	863	3	8	13
1313	1651	552	2	8	14
6698	1288	414	1	8	14
-	1564	575	2	11	14
-	4300	552	3	9	14
-	1196	713	2	9	14
-	1121	462	1	9	14
-	1459	736	1	13	14
-	1266	575	1	15	14
-	3337	667	2	15	14
-	1805	437	2	13	-
-	1520	460	2	12	-
-	3245	575	2	12	-
-	1207	484	2	10	-
-	1138	989	1	10	-
-	834	575	1	11	-
-	1230	621	1	-	-
-	2277	531	1	-	-
-	3070	414	1	-	-
-	1564	437	1	-	-
-	4300	690	1	-	-
-	1196	690	1	-	-
-	1121	598	1	-	-
-	1459	462	1	-	-
-	2342	805	1	-	-
-	2208	368	1	-	-
-	2208	368	-	-	-
-	1584	293	-	-	-
-	1983	776	-	-	-
-	1452	863	-	-	-
-	1138	414	-	-	-
-	1130	466	-	-	-
-	1651	535	-	-	-
-	1288	587	-	-	-

-	1564	466	-	-	-
-	-	828	-	-	-
-	-	667	-	-	-
-	-	736	-	-	-
-	-	598	-	-	-
-	-	759	-	-	-
-	-	369	-	-	-
-	-	598	-	-	-
-	-	782	-	-	-
-	-	462	-	-	-
-	-	530	-	-	-
-	-	667	-	-	-
-	-	805	-	-	-
-	-	690	-	-	-
-	-	736	-	-	-
-	-	506	-	-	-
-	-	623	-	-	-
-	-	667	-	-	-
-	-	989	-	-	-

CCV measures									
Figure 2									
CCV size (nm)					n° CCV/Cell				
EGFP	HDAC6 WT	HDAC6 H216A/H611A	αTAT WT	αTAT D157N	EGFP	HDAC6 WT	HDAC6 H216A/H611A	αTAT WT	αTAT D157N
6600	484	14460	2587	3967	1	22	1	3	30
7040	794	9695	4140	1224	1	20	1	3	30
4400	828	6097	5012	1580	1	23	1	3	30
4400	484	5914	6472	3808	1	23	2	1	30
9020	725	9576	1288	875	2	23	2	3	30
6930	725	5754	3496	1426	2	23	2	3	30
4785	759	7772	3864	1196	4	23	2	3	30
10920	621	10854	1978	874	2	23	2	3	30
11060	690	15872	7660	1449	1	21	1	3	30
19371	414	6934	7012	3753	2	21	2	1	30
7606	621	15321	7314	1794	2	21	2	2	30
13780	690	18936	6678	7797	3	21	3	1	30
6760	656	9294	5951	2530	3	21	3	2	30
10920	1105	7081	2355	1794	2	21	2	3	31
7800	622	5658	2293	1564	1	30	1	2	32
3380	690	4272	3797	1518	2	30	2	3	32
6240	391	7499	5955	1288	3	30	3	5	36
7020	461	13259	6081	1564	2	29	2	3	31
9100	392	9420	4968	4300	1	29	1	5	30
8840	345	12883	4140	1196	2	25	2	5	29
8580	575	9950	2518	4209	3	28	3	5	28
12740	484	8384	6680	2967	2	28	1	4	28
17420	644	11558	6463	3691	1	26	1	4	28
5280	299	17993	9936	2684	2	25	1	5	29
13400	368	20495	5521	1932	3	26	1	5	29
7040	276	11946	6578	1426	2	25	1	3	29
7530	138	6700	5474	1104	2	26	2	3	24
8100	621	8856	3337	5037	4	26	1	3	25

5520	299	7793	4805	1173	4	26	4	2	30
5107	231	11930	5520	966	2	26	2	1	30
4821	230	11167	3245	1112	1	26	1	5	30
5000	621	20200	9207	1588	1	26	1	5	30
4985	553	7784	2967	8287	1	26	1	3	30
6500	311	8839	1969	794	1	26	1	5	30
18070	519	11234	2145	1552	1	26	1	4	30
16315	484	9893	2484	1932	4	26	4	4	30
13100	449	11193	8759	1587	4	26	4	1	28
6810	829	1294	8690	4332	4	26	4	2	28
12480	588	7081	1173	873	4	26	4	3	28
8580	759	5658	1600	763	4	30	2	5	28
8970	461	4272	1518	1658	4	30	2	5	28
9346	511	7499	5523	4076	2	30	2	5	28
8377	484	13259	4795	6693	2	30	2	5	28
19000	414	9420	4347	6625	1	27	1	4	23
6300	322	12883	2208	3036	2	27	2	5	25
7939	369	9950	2208	899	2	27	2	2	26
6675	230	-	1864	1242	2	27	2	3	21
8690	531	-	5799	1243	2	20	2	3	32
7559	530	-	1524	875	2	18	1	3	37
5934	484	-	1656	1870	2	18	2	3	38
9380	531	-	2576	4370	2	20	1	3	32
9004	621	-	2530	2518	1	20	1	3	32
8122	485	-	8702	2346	1	21	1	3	30
6391	673	-	5060	2036	1	21	1	2	30
7473	442	-	4416	3555	1	23	1	2	32
6500	575	-	3082	4589	1	25	1	2	30
6820	667	-	6380	3174	1	25	1	1	29
11330	944	-	3220	2139	2	26	2	1	29
6600	415	-	2070	9246	2	29	2	1	-
7263	552	-	10905	248	1	29	3	2	-
11770	644	-	7453	2346	2	30	3	3	-
5445	345	-	8142	2277	2	28	2	3	-

8500	276	-	3461	3070	3	28	1	2	-
7533	552	-	6348	2001	2	28	2	2	-
12480	368	-	4969	1966	1	28	1	1	-
-	322	-	4418	1733	2	28	2	1	-
-	829	-	4418	2141	3	28	3	1	-
-	967	-	1380	1566	2	28	1	2	-
-	600	-	10626	1898	1	28	1	3	-
-	598	-	3180	1829	1	28	2	3	-
-	484	-	2002	2277	1	28	2	-	-
-	345	-	3450	2070	2	32	2	-	-
-	575	-	6285	-	2	32	2	-	-
-	782	-	8278	-	2	32	2	-	-
-	506	-	4558	-	2	-	2	-	-
-	277	-	6141	-	2	-	2	-	-
-	345	-	-	-	1	-	1	-	-
-	380	-	-	-	1	-	1	-	-
-	519	-	-	-	1	-	1	-	-
-	311	-	-	-	1	-	1	-	-
-	380	-	-	-	1	-	1	-	-
-	345	-	-	-	1	-	1	-	-
-	386	-	-	-	1	-	2	-	-
-	345	-	-	-	1	-	2	-	-
-	380	-	-	-	1	-	2	-	-
-	483	-	-	-	1	-	3	-	-
-	415	-	-	-	1	-	1	-	-
-	552	-	-	-	1	-	1	-	-
-	483	-	-	-	1	-	1	-	-
-	518	-	-	-	1	-	1	-	-
-	483	-	-	-	1	-	1	-	-
-	552	-	-	-	1	-	-	-	-
-	449	-	-	-	1	-	-	-	-
-	518	-	-	-	1	-	-	-	-
-	587	-	-	-	1	-	-	-	-
-	345	-	-	-	1	-	-	-	-

-	760	-	-	-	-	-	-	-	-
-	345	-	-	-	-	-	-	-	-
-	454	-	-	-	-	-	-	-	-
-	381	-	-	-	-	-	-	-	-
-	345	-	-	-	-	-	-	-	-
-	484	-	-	-	-	-	-	-	-
-	380	-	-	-	-	-	-	-	-
-	483	-	-	-	-	-	-	-	-
-	519	-	-	-	-	-	-	-	-
-	415	-	-	-	-	-	-	-	-
-	834	-	-	-	-	-	-	-	-
-	415	-	-	-	-	-	-	-	-
-	415	-	-	-	-	-	-	-	-
-	794	-	-	-	-	-	-	-	-

CCV measures											
Figure 3											
CCV size (nm)						n° CCV/Cell					
EGFP	p150 WT	p50 WT	p150 CC1	RILP WT	RILP ΔN	EGFP	p150 WT	p50 WT	p150 CC1	RILP WT	RILP ΔN
1102	5517	311	380	1794	1127	1	1	22	30	3	15
2320	5282	415	318	8000	3375	1	1	20	30	3	15
8990	8747	829	552	2625	1583	1	1	23	30	1	15
9034	3450	621	381	7081	1172	1	2	23	30	5	15
7834	7314	173	276	3427	1923	2	2	23	30	5	15
6496	2487	138	414	6432	1255	2	2	23	30	3	15
8404	1518	483	494	2246	1391	2	2	23	28	5	18
8734	1794	415	591	5810	3485	1	2	23	28	4	18
5632	8000	318	207	1661	1311	2	1	21	28	4	18
2003	2625	759	312	3174	1214	3	2	21	28	1	18
6407	7081	380	218	2979	1943	2	2	21	28	2	18
8904	3427	519	207	9585	2741	1	3	21	28	3	18
7839	6432	312	176	5704	1130	2	3	21	28	5	18
8934	2246	414	311	5282	1651	3	2	21	23	3	18
7956	5810	552	173	8747	801	1	1	20	32	2	18
1002	1661	311	251	3450	3017	1	2	20	32	3	18
2324	3174	454	276	7314	1278	1	3	21	36	5	18
6723	2979	450	552	2487	842	2	2	21	31	3	18
5626	9585	414	311	1518	2146	1	1	23	30	5	18
11102	5704	347	454	1794	1113	2	2	25	29	5	18
4340	8331	518	450	8000	1125	3	3	25	28	3	18
8902	3048	278	414	2625	663	2	1	26	28	5	12
7828	11851	380	347	7081	1484	1	1	29	28	5	12
7882	6875	345	518	3427	1983	2	1	25	29	5	12
9090	1542	587	278	6432	1632	3	1	26	29	5	12
10232	7739	450	380	2246	1138	2	1	25	29	4	12
2325	2410	312	345	2625	1834	2	2	26	24	5	12
7893	7640	276	587	7081	1220	4	1	26	25	3	12
6749	11401	771	450	3427	2277	4	4	26	30	2	12
9983	3951	381	312	6432	3070	2	2	26	30	1	12

10223	3518	138	276	2246	3001	1	1	26	30	5	12
2321	3369	415	771	5810	1966	1	1	26	30	5	12
1203	4058	276	381	1661	1733	1	1	26	30	3	21
5632	9739	386	138	3174	2141	1	1	26	30	5	21
8580	-	347	415	3369	1566	1	1	26	30	4	20
8970	-	380	311	4058	1496	4	4	26	30	4	20
7346	-	347	415	9739	1139	4	4	26	28	1	20
8377	-	345	829	5456	1128	2	4	26	28	2	20
9000	-	380	621	3048	1346	3	4	26	28	3	23
6300	-	318	173	5566	3900	3	2	30	28	5	14
7939	-	552	138	1203	2994	2	2	28	28	5	15
18720	-	381	483	5632	1104	1	2	28	28	5	16
4800	-	276	415	8580	3456	2	2	28	28	5	16
8840	-	414	318	8970	3875	3	1	28	23	4	16
9004	-	494	759	7346	9543	2	2	28	25	5	18
9122	-	591	380	8377	1234	1	2	28	26	2	18
6391	-	207	519	-	1124	2	2	28	21	3	18
7473	-	312	312	-	1834	3	2	28	32	3	20
8500	-	218	414	-	1145	2	1	18	37	3	-
6820	-	207	552	-	1314	1	2	18	38	3	-
11330	-	176	484	-	1673	2	1	20	32	3	-
-	-	311	347	-	3499	3	1	20	25	3	-
-	-	173	484	-	1398	2	1	21	26	4	-
-	-	251	449	-	1566	1	1	21	25	4	-
-	-	276	311	-	1898	2	1	23	26	4	-
-	-	552	449	-	-	3	1	25	26	4	-
-	-	311	176	-	-	1	1	25	26	4	-
-	-	454	347	-	-	1	2	26	26	2	-
-	-	450	312	-	-	1	2	29	26	4	-
-	-	414	552	-	-	1	3	29	26	4	-
-	-	347	794	-	-	1	3	30	30	4	-
-	-	518	488	-	-	1	2	28	30	4	-
-	-	278	519	-	-	4	1	28	30	-	-
-	-	380	311	-	-	4	2	28	30	-	-

-	-	345	414	-	-	4	1	28	28	-	-
-	-	587	588	-	-	4	2	28	28	-	-
-	-	450	483	-	-	2	3	28	28	-	-
-	-	312	488	-	-	2	1	28	28	-	-
-	-	276	2316	-	-	2	1	28	28	-	-
-	-	771	1380	-	-	2	2	28	28	-	-
-	-	381	483	-	-	1	2	28	28	-	-
-	-	138	483	-	-	2	2	32	23	-	-
-	-	415	483	-	-	2	2	32	-	-	-
-	-	276	347	-	-	2	2	32	-	-	-
-	-	386	345	-	-	2	2	-	-	-	-
-	-	138	552	-	-	2	2	-	-	-	-
-	-	415	553	-	-	1	1	-	-	-	-
-	-	276	312	-	-	1	1	-	-	-	-
-	-	386	380	-	-	1	1	-	-	-	-
-	-	347	552	-	-	1	1	-	-	-	-
-	-	380	417	-	-	-	1	-	-	-	-
-	-	347	322	-	-	-	1	-	-	-	-
-	-	345	414	-	-	-	2	-	-	-	-
-	-	368	235	-	-	-	2	-	-	-	-
-	-	322	417	-	-	-	2	-	-	-	-
-	-	414	414	-	-	-	3	-	-	-	-
-	-	692	325	-	-	-	1	-	-	-	-
-	-	424	469	-	-	-	1	-	-	-	-
-	-	598	552	-	-	-	1	-	-	-	-
-	-	879	598	-	-	-	1	-	-	-	-
-	-	1426	322	-	-	-	1	-	-	-	-
-	-	605	414	-	-	-	-	-	-	-	-
-	-	1104	417	-	-	-	-	-	-	-	-
-	-	1841	508	-	-	-	-	-	-	-	-
-	-	787	737	-	-	-	-	-	-	-	-
-	-	749	1748	-	-	-	-	-	-	-	-
-	-	696	2760	-	-	-	-	-	-	-	-
-	-	514	280	-	-	-	-	-	-	-	-

-	-	644	230	-	-	-	-	-	-	-	-	-
-	-	692	514	-	-	-	-	-	-	-	-	-
-	-	614	368	-	-	-	-	-	-	-	-	-
-	-	600	598	-	-	-	-	-	-	-	-	-
-	-	1173	644	-	-	-	-	-	-	-	-	-
-	-	1104	414	-	-	-	-	-	-	-	-	-
-	-	1104	514	-	-	-	-	-	-	-	-	-
-	-	828	230	-	-	-	-	-	-	-	-	-
-	-	2070	280	-	-	-	-	-	-	-	-	-
-	-	4004	417	-	-	-	-	-	-	-	-	-
-	-	414	483	-	-	-	-	-	-	-	-	-
-	-	138	690	-	-	-	-	-	-	-	-	-
-	-	483	506	-	-	-	-	-	-	-	-	-
-	-	900	506	-	-	-	-	-	-	-	-	-
-	-	3036	368	-	-	-	-	-	-	-	-	-
-	-	762	414	-	-	-	-	-	-	-	-	-
-	-	276	184	-	-	-	-	-	-	-	-	-
-	-	414	371	-	-	-	-	-	-	-	-	-
-	-	449	322	-	-	-	-	-	-	-	-	-
-	-	449	2300	-	-	-	-	-	-	-	-	-
-	-	518	230	-	-	-	-	-	-	-	-	-
-	-	380	322	-	-	-	-	-	-	-	-	-
-	-	-	145	-	-	-	-	-	-	-	-	-
-	-	-	248	-	-	-	-	-	-	-	-	-
-	-	-	1979	-	-	-	-	-	-	-	-	-
-	-	-	976	-	-	-	-	-	-	-	-	-
-	-	-	322	-	-	-	-	-	-	-	-	-
-	-	-	569	-	-	-	-	-	-	-	-	-
-	-	-	379	-	-	-	-	-	-	-	-	-
-	-	-	552	-	-	-	-	-	-	-	-	-
-	-	-	1887	-	-	-	-	-	-	-	-	-
-	-	-	393	-	-	-	-	-	-	-	-	-
-	-	-	1058	-	-	-	-	-	-	-	-	-
-	-	-	414	-	-	-	-	-	-	-	-	-

-	-	-	646	-	-	-	-	-	-	-	-
-	-	-	1311	-	-	-	-	-	-	-	-
-	-	-	771	-	-	-	-	-	-	-	-
-	-	-	488	-	-	-	-	-	-	-	-
-	-	-	693	-	-	-	-	-	-	-	-
-	-	-	2829	-	-	-	-	-	-	-	-
-	-	-	414	-	-	-	-	-	-	-	-
-	-	-	138	-	-	-	-	-	-	-	-
-	-	-	345	-	-	-	-	-	-	-	-
-	-	-	483	-	-	-	-	-	-	-	-
-	-	-	345	-	-	-	-	-	-	-	-
-	-	-	436	-	-	-	-	-	-	-	-
-	-	-	345	-	-	-	-	-	-	-	-
-	-	-	488	-	-	-	-	-	-	-	-
-	-	-	552	-	-	-	-	-	-	-	-
-	-	-	2763	-	-	-	-	-	-	-	-
-	-	-	322	-	-	-	-	-	-	-	-

CCV measures											
Figure 4											
CCV size (nm)						n° CCV/Cell					
EGFP	RILP WT + Rab7 WT	RILP ΔN + Rab7 WT	RILP WT + p150 WT	RILP ΔN + p150 WT	RILP WT + p50 WT	EGFP	RILP WT + Rab7 WT	RILP ΔN + Rab7 WT	RILP WT + p150 WT	RILP ΔN + p150 WT	RILP WT + p50 WT
5829	4102	2008	6496	1024	1923	3	3	22	3	17	12
2277	6320	1224	8404	1034	2556	3	3	20	3	18	12
2070	8990	995	8734	2012	834	3	1	23	3	17	13
8823	9034	1600	5632	1346	1220	3	5	29	3	17	13
8556	7834	3045	2003	1923	2277	3	5	29	3	18	14
2694	6496	2112	6407	2556	3070	3	3	29	3	20	12
5001	8404	1024	8904	834	2001	3	5	29	3	20	14
6663	8734	1034	7839	1220	1966	3	4	29	3	23	13
2584	5632	2012	8934	2277	1733	3	2	29	3	23	14
8983	2003	1346	7956	3070	2141	3	2	29	3	23	14
5452	6407	1923	6002	2001	566	2	2	29	2	23	14
6138	8904	2556	4102	1966	3070	3	2	29	3	23	12
10834	7839	834	6320	1733	2001	3	2	29	6	23	12
6230	8934	1220	8990	2141	1966	2	2	29	6	23	14
8277	7956	2277	9034	566	733	1	2	29	6	23	14
3070	6002	3070	7834	3070	1141	2	2	29	6	23	13
3601	2324	2001	6496	2001	7870	3	2	21	6	23	15
8234	6723	1966	2325	1966	1298	2	2	21	6	23	15
9975	5626	1733	7893	733	1243	1	2	23	6	23	15
4244	8102	2141	6749	1141	1875	2	2	25	6	23	17
5632	4340	566	9983	7870	1922	3	3	25	3	23	17
6342	8902	3070	4223	1298	470	1	5	26	3	23	15
1168	7828	2001	2321	1243	518	1	5	29	3	23	14
4318	7882	1966	6203	1012	236	1	5	25	3	21	12
3379	9090	733	5632	1275	578	1	5	24	3	21	13
2563	6232	1141	9975	1426	1521	1	4	24	3	23	13
8196	2325	7870	4244	1104	2520	2	5	24	3	25	13
9943	7893	1298	5632	874	1337	1	3	24	3	25	14
11283	6749	1243	6342	1104	1245	4	3	24	3	26	14

4134	9983	1875	1168	1242	1805	2	3	24	3	29	14
6988	4223	1922	4318	3966	1680	1	3	24	3	25	15
5231	2321	470	8556	2438	1474	1	3	24	3	24	15
-	6203	518	2694	1242	1463	1	3	24	3	24	16
-	5632	1346	5001	1104	2520	1	5	24	3	24	11
-	-	1552	-	1661	1337	1	3	24	3	24	11
-	-	693	-	1245	1245	4	4	26	3	24	11
-	-	125	-	2001	1805	4	1	26	3	24	10
-	-	336	-	1966	1680	4	2	26	2	24	10
-	-	899	-	733	1474	4	3	26	2	24	10
-	-	1242	-	1141	1463	2	5	30	2	24	12
-	-	236	-	7870	2207	2	5	28	2	24	15
-	-	578	-	1298	1578	2	5	28	2	24	15
-	-	1521	-	1243	1288	2	5	28	1	26	12
-	-	2520	-	1875	1564	1	4	28	1	26	10
-	-	1337	-	1922	1300	2	5	28	1	21	12
-	-	1245	-	470	1196	2	2	28	4	21	14
-	-	1805	-	-	1463	2	3	28	4	23	14
-	-	1680	-	-	2207	2	3	28	4	25	13
-	-	1474	-	-	1578	1	3	18	4	25	15
-	-	1463	-	-	1288	2	3	18	2	26	15
-	-	2207	-	-	3070	1	3	20	2	29	15
-	-	1578	-	-	2001	1	3	20	2	25	17
-	-	1288	-	-	1966	1	4	21	2	24	17
-	-	1564	-	-	1733	1	4	21	1	24	15
-	-	1300	-	-	2141	1	4	23	2	24	14
-	-	1196	-	-	566	1	4	25	2	24	12
-	-	1121	-	-	1012	1	4	24	2	24	-
-	-	1459	-	-	-	2	2	24	2	24	-
-	-	1266	-	-	-	2	4	24	1	24	-
-	-	1012	-	-	-	3	4	24	2	24	-
-	-	1275	-	-	-	3	4	24	1	21	-
-	-	1426	-	-	-	2	4	24	1	21	-
-	-	1104	-	-	-	1	-	24	1	21	-

-	-	874	-	-	-	2	-	24	-	21	-
-	-	1104	-	-	-	1	-	24	-	21	-
-	-	1242	-	-	-	2	-	24	-	21	-
-	-	3966	-	-	-	3	-	28	-	21	-
-	-	2438	-	-	-	1	-	28	-	21	-
-	-	1242	-	-	-	1	-	28	-	-	-
-	-	1104	-	-	-	2	-	28	-	-	-
-	-	1661	-	-	-	2	-	28	-	-	-
-	-	1245	-	-	-	2	-	-	-	-	-
-	-	1472	-	-	-	2	-	-	-	-	-
-	-	364	-	-	-	2	-	-	-	-	-

CCV measures									
Figure 5									
CCV size (nm)					n° CCV/Cell				
EGFP	KIF5 WT	KIF5 T93N	FYCO1 WT	αTAT Δ555-1136	EGFP	KIF5 WT	KIF5 T93N	FYCO1 WT	αTAT Δ555-1136
7690	1966	487	3079	1345	1	3	1	3	1
5805	6733	759	5598	339	1	3	1	3	1
2422	2141	1345	5691	121	1	1	1	3	1
4075	1566	2314	3913	2173	1	5	1	3	1
3085	6898	3450	3836	1207	2	5	2	3	2
4649	1829	552	1449	223	2	3	2	3	2
9112	2277	1249	4508	552	4	5	4	3	4
7324	2070	1553	5828	1587	2	4	2	3	2
12420	6823	3149	5828	621	1	2	1	3	1
5460	8556	514	1112	437	2	2	2	3	2
9695	2694	1279	5553	2582	3	2	2	2	2
6097	4830	946	4278	449	1	2	3	3	3
4914	4526	1599	1318	220	1	2	3	6	1
10576	4075	2781	1249	207	1	2	2	6	2
5754	1004	1726	4925	1288	1	2	1	6	2
3772	6582	351	1037	1386	1	2	2	6	4
10854	7565	1462	1037	223	2	2	3	6	2
15872	6239	593	6828	2278	1	2	2	6	1
6934	1518	419	4291	453	4	2	1	6	2
5321	8045	2345	1339	554	2	2	2	6	2
8936	1904	308	4602	2967	1	3	3	3	3
11294	1098	1380	1002	1381	1	5	2	3	3
7081	859	741	4998	2492	1	5	1	3	2
5658	3552	1368	3993	452	1	5	2	3	1
5272	6506	833	2203	223	1	5	3	3	2
7499	1010	276	4752	2058	4	4	2	3	3
3259	6426	3337	4099	2426	4	5	2	3	2
9420	6966	1552	4647	674	4	3	4	3	1
3197	4828	502	2005	1469	4	3	4	3	2

7472	7586	552	1805	1455	2	3	2	3	3
4212	5107	414	6949	1395	2	3	1	3	2
5244	2346	1345	2995	1730	2	3	1	3	1
7384	1518	1351	2447	2088	2	3	1	3	2
6900	2088	218	2636	423	1	5	1	3	3
4416	1359	502	1658	331	2	3	1	3	2
4570	4407	1944	3795	874	2	4	4	3	2
3923	5684	1703	6184	2043	2	1	4	3	4
-	1706	636	4000	1381	2	2	4	2	4
-	2911	1276	7966	1826	1	3	4	2	2
-	8743	414	3736	2682	2	5	4	2	1
-	3036	414	4076	1164	1	5	4	2	1
-	1748	1284	1600	276	1	5	2	2	1
-	1182	436	4070	1150	1	5	2	1	1
-	6104	1864	2300	2322	1	4	1	1	1
-	1150	436	4011	1123	1	5	2	1	4
-	6462	1953	2003	1242	1	2	2	4	4
-	2622	2217	4568	279	1	3	2	4	4
-	6689	234	5191	554	2	3	2	4	4
-	5663	2184	1874	2178	2	3	2	4	4
-	2625	2184	4019	1055	3	3	2	2	4
-	1600	1379	2501	1661	3	3	2	2	2
-	9966	762	2403	414	2	3	1	2	2
-	1272	1975	-	502	1	4	1	2	1
-	1181	718	-	828	2	4	1	1	2
-	6829	645	-	483	1	4	1	2	2
-	2830	334	-	419	2	4	1	2	2
-	8924	1696	-	1656	3	4	1	2	2
-	1150	308	-	1243	1	2	2	2	2
-	-	2102	-	207	1	4	2	1	2
-	-	1350	-	759	2	4	4	2	2
-	-	737	-	483	2	4	2	1	1
-	-	2276	-	3797	2	6	1	1	1
-	-	1325	-	1035	2	6	2	3	1

-	-	1462	-	552	2	6	3	3	1
-	-	416	-	830	2	6	1	3	1
-	-	393	-	436	2	6	1	3	1
-	-	325	-	828	1	6	1	3	2
-	-	2106	-	762	1	6	1	4	2
-	-	508	-	1112	1	3	1	4	4
-	-	325	-	1936	1	3	2	4	2
-	-	3184	-	621	1	3	1	4	1
-	-	960	-	345	2	3	4	4	2
-	-	1650	-	2537	2	3	2	2	3
-	-	1184	-	2075	1	3	1	4	1
-	-	247	-	1205	2	3	1	4	1
-	-	145	-	1794	1	3	1	4	2
-	-	4752	-	853	1	3	1	-	2
-	-	1276	-	621	1	3	1	-	2
-	-	379	-	966	1	3	4	-	2
-	-	1234	-	-	1	3	4	-	2
-	-	436	-	-	1	3	4	-	2
-	-	469	-	-	1	-	4	-	2
-	-	1508	-	-	2	-	2	-	1
-	-	414	-	-	2	-	2	-	3
-	-	3252	-	-	3	-	2	-	1
-	-	1552	-	-	3	-	2	-	1
-	-	416	-	-	2	-	1	-	1
-	-	1184	-	-	1	-	2	-	1
-	-	460	-	-	2	-	2	-	1
-	-	1370	-	-	1	-	-	-	1
-	-	1145	-	-	2	-	-	-	1
-	-	-	-	-	3	-	-	-	1
-	-	-	-	-	1	-	-	-	1
-	-	-	-	-	-	-	-	-	1
-	-	-	-	-	-	-	-	-	1
-	-	-	-	-	-	-	-	-	1

CCV measures											
Figure 6											
CCV size (nm)						n° CCV/Cell					
EGFP	hVps41 WT	hVps41 A187T	hVps41 WT + RILP WT	hVps41 WT + RILP ΔN	hVps41 A187T + RILP WT	EGFP	hVps41 WT	hVps41 A187T	hVps41 WT + RILP WT	hVps41 WT + RILP ΔN	hVps41 A187T + RILP WT
14460	1746	2587	9112	1127	1983	2	3	20	1	24	23
9695	11040	4140	7324	3375	1452	1	1	20	1	24	23
6097	9448	1012	1420	1583	1138	2	2	20	2	24	23
5914	9112	1472	3460	1172	834	3	1	20	1	24	23
1576	20324	1288	9695	1923	1230	1	2	20	2	24	23
5754	12420	3496	6097	1255	2277	2	3	20	1	24	23
3772	23460	3864	1914	1391	3070	3	1	20	2	24	23
10854	9695	1978	3576	3485	3601	4	2	20	3	24	23
15872	6097	760	5754	1311	1234	5	3	20	1	24	23
6934	14914	1012	3772	1214	9975	1	4	20	2	24	26
15321	4700	2314	1854	1943	1244	2	1	23	1	27	26
18936	6905	1678	872	2741	5632	3	2	23	2	27	26
1294	7197	1951	6934	1130	2342	1	1	23	3	27	26
7081	17628	2355	5321	1651	1168	2	2	25	1	27	26
5658	5272	2293	8936	801	2318	3	3	25	1	27	26
4272	8284	3797	1294	3017	3379	1	1	25	2	27	30
7499	4692	2955	7081	1278	2563	2	2	26	1	27	30
13259	11319	1081	5658	9842	1196	3	1	24	2	27	30
9420	14564	4968	5272	2146	9943	4	2	25	1	27	30
12883	4975	4140	7499	1113	1283	2	3	25	1	27	29
9950	5796	2518	3259	5001	1134	2	1	25	2	27	29
8384	7497	1680	9420	8663	988	1	2	24	3	27	29
11558	3499	1463	7197	1484	1231	2	1	25	1	27	29
17993	5773	9936	7472	1983	3967	3	2	28	1	27	29
20495	10795	5521	2212	1632	1224	1	3	28	2	27	29
11946	7747	6578	5244	1138	1580	2	4	29	3	27	29
6700	6348	1474	7384	1834	6808	3	1	29	1	27	28
8856	8298	3337	6900	1220	875	4	2	25	2	27	28

7793	11259	1805	4416	2277	1426	1	3	25	3	24	28
11930	9535	5520	1570	3070	1196	2	4	28	1	23	28
11167	12407	3245	1488	3001	874	3	5	23	1	23	28
20200	11095	1207	8832	1966	1449	4	3	23	2	23	28
7784	5272	2967	4418	1733	8753	1	1	20	1	23	28
8839	7499	1969	4418	2141	1794	2	2	23	2	23	28
11234	13259	2145	1380	1566	7797	3	3	23	3	29	31
9893	9420	2484	1626	1496	2530	4	1	23	1	28	31
11193	7197	759	3180	5339	1794	5	2	23	1	28	31
1294	7472	690	2002	1128	1564	2	3	23	2	28	24
7081	10212	1173	1450	4346	1518	1	1	23	1	28	24
5658	5244	1600	6285	3900	1288	1	2	23	2	28	24
4272	7384	1518	1278	2994	1564	2	3	23	3	28	24
7499	6900	5523	1558	1104	4300	3	4	23	1	28	24
13259	4416	4795	2141	3456	1196	4	1	23	2	28	24
9420	14570	4347	7870	3875	4209	1	2	23	1	28	24
12883	10488	2208	4370	9543	2967	2	1	23	1	24	24
9950	8832	2208	2518	1234	3691	3	2	23	2	24	24
8840	12325	1864	2346	1124	1684	4	3	23	1	24	24
8580	11043	5799	2036	1834	1932	5	1	23	2	24	24
12740	8836	1524	3555	1145	1426	3	2	23	1	24	24
17420	7181	1656	4589	1314	1104	1	3	23	2	29	26
5280	9400	2576	3174	1673	6037	2	4	23	3	29	26
13400	12974	2530	2139	3499	1173	3	5	23	1	29	26
7040	17388	1702	1246	1398	966	1	3	23	2	29	26
7530	14078	5060	1248	1566	1112	2	1	23	3	29	26
8100	9695	4416	2346	1898	1588	3	2	23	1	29	26
5520	11316	3082	2277	1829	8287	4	3	23	1	29	26
5107	5251	1380	3070	2277	794	5	4	23	2	29	26
4821	9939	3220	2001	2070	1552	4	5	23	1	29	26
5000	11868	2070	1966	8823	1932	7	1	23	2	30	26
4985	8556	1905	1733	8556	1587	1	2	23	1	30	26
12420	11595	7453	2141	2694	1332	2	3	23	2	30	26
23460	8560	8142	1566	5001	873	3	4	23	1	30	26

9695	-	3461	1898	6663	763	1	1	23	2	30	26
6097	-	6348	1829	-	1658	2	2	23	3	30	26
14914	-	4969	2277	-	1076	3	3	23	1	26	26
4700	-	-	2070	-	6693	4	1	23	1	26	26
6905	-	-	2823	-	6625	5	2	23	2	26	26
7197	-	-	2556	-	3036	1	3	23	3	26	26
17628	-	-	2694	-	899	1	1	23	4	26	27
5272	-	-	4830	-	1242	1	2	23	1	26	27
-	-	-	1526	-	1243	1	3	22	2	26	27
-	-	-	4075	-	1875	1	4	22	3	26	27
-	-	-	1004	-	-	2	5	22	1	26	27
-	-	-	2582	-	-	3	1	22	2	26	27
-	-	-	4565	-	-	1	2	22	1	-	27
-	-	-	6239	-	-	2	1	22	2	-	27
-	-	-	1518	-	-	3	2	22	3	-	27
-	-	-	8045	-	-	1	3	25	4	-	-
-	-	-	1904	-	-	2	4	25	1	-	-
-	-	-	3098	-	-	3	1	25	1	-	-
-	-	-	-	-	-	2	2	25	2	-	-
-	-	-	-	-	-	3	3	25	1	-	-
-	-	-	-	-	-	4	4	25	2	-	-
-	-	-	-	-	-	5	1	25	3	-	-
-	-	-	-	-	-	1	2	25	-	-	-
-	-	-	-	-	-	2	3	-	-	-	-
-	-	-	-	-	-	1	4	-	-	-	-
-	-	-	-	-	-	-	5	-	-	-	-

CCV measures											
Figure 7											
CCV size (nm)						n° CCV/Cell					
EGFP	Arl8 WT	Arl8 T34N	hVps41 WT + Arl8 WT	hVps41 WT + Arl8 T34N	hVps41 A187T + Arl8 WT	EGFP	Arl8 WT	Arl8 T34N	hVps41 WT + Arl8 WT	hVps41 WT + Arl8 T34N	hVps41 A187T + Arl8 WT
7666	8832	1657	6578	1390	5219	1	1	20	1	24	22
11040	12325	2208	5521	2019	4795	2	2	21	1	24	25
8448	11043	2212	5520	3427	4347	3	3	21	2	32	23
9112	8836	1035	3337	1572	2208	1	4	21	3	32	24
6324	7181	621	1245	3304	2208	2	5	21	4	32	24
12420	9400	1106	1805	4301	1584	3	6	20	5	32	24
4460	7974	2760	1680	2210	1983	4	1	23	1	32	30
9695	7388	2626	1474	7002	1452	5	2	23	2	32	29
6097	7078	2286	1463	1993	1138	1	3	23	3	32	29
8445	9695	4969	2207	8449	834	2	4	23	1	32	29
4700	7316	4418	4578	1142	1230	3	5	23	2	27	28
6905	5251	4418	1288	5009	2277	1	6	23	1	27	28
7197	9939	1380	1564	6503	3070	2	7	23	2	27	24
7628	17628	1626	4300	3453	3601	3	1	23	3	27	24
5272	5272	3180	1196	2324	1234	1	2	25	4	27	24
8690	8284	1288	1121	4568	9975	2	3	25	1	27	29
3559	4692	1456	1459	930	1244	3	4	26	2	27	29
6865	11319	542	1266	7382	5632	4	5	24	1	27	29
5041	14564	2070	3337	5839	2342	5	1	24	2	27	29
5226	4975	1690	1805	6503	1168	6	2	24	3	27	29
5520	5796	1183	5520	2210	1583	1	3	24	4	27	29
5107	7497	1387	3245	1023	1172	2	4	24	5	27	29
4821	3499	1815	1207	1430	1923	3	5	24	6	30	29
5272	5773	2713	2967	4530	1255	1	6	24	1	30	29
7499	10795	3500	1969	6775	1391	2	7	24	2	30	29
13259	7747	5698	2145	3537	6578	3	8	24	3	30	29
9420	6348	5003	1518	1004	5521	4	1	24	4	30	28
7197	8298	4256	1380	7843	5520	1	2	25	5	30	28

7472	11259	1679	568	1223	3337	2	3	25	1	30	28
10212	9535	2198	6578	1022	1245	3	1	18	2	30	28
5244	12407	722	1474	4304	1805	4	2	18	3	30	28
3183	11095	657	7954	2024	1680	1	3	18	4	30	28
6180	5272	2831	5219	2201	1474	2	4	20	5	30	28
7000	7499	2014	4795	4305	1463	3	5	23	1	23	28
3079	13259	2300	4347	9902	2207	4	1	23	2	29	31
5658	9420	5658	2208	1120	4578	5	2	23	3	28	31
4761	7197	4761	2208	2001	1288	6	3	23	1	28	31
3625	7472	3625	1584	2920	1564	1	4	23	2	28	24
5951	10212	1951	1983	-	4300	1	5	23	3	28	24
5041	5244	5041	1452	-	1196	2	7	23	4	28	24
3492	7384	3492	1138	-	1121	3	1	23	5	29	24
7954	6900	3337	834	-	1459	4	2	23	6	29	24
5219	5796	1805	1230	-	1805	1	3	23	1	29	24
2480	7497	520	2277	-	520	2	4	19	2	29	24
8580	3499	3245	3070	-	3245	3	5	23	3	29	24
8970	5773	714	3601	-	714	4	1	23	4	24	24
1346	10795	2139	1234	-	2139	5	2	23	1	24	25
8377	7747	3542	9975	-	3542	6	3	23	2	24	25
9000	6348	4278	1244	-	4278	1	4	23	3	24	25
6300	8298	966	5632	-	966	2	5	23	1	29	25
7939	6259	1295	2342	-	1295	3	1	23	2	29	25
5219	9535	1288	1168	-	1288	1	2	22	3	29	25
2480	9407	2553	1583	-	2553	2	3	22	1	29	25
8580	14078	2346	1172	-	2346	3	4	22	2	29	25
8970	9695	1311	1923	-	2277	4	5	22	3	24	26
1346	11316	1519	1255	-	3070	5	6	22	4	24	26
8377	5251	1472	1391	-	3601	6	7	22	1	24	26
-	9939	1518	3485	-	1234	7	1	22	2	24	26
-	11868	1380	1311	-	9975	1	2	22	3	24	26
-	8556	568	1214	-	1244	2	3	23	1	24	26
-	11595	6578	8943	-	5632	3	4	23	2	30	26
-	8560	1474	2741	-	2342	1	5	23	1	30	26

-	-	754	1130	-	1168	2	1	23	2	30	26
-	-	5219	1651	-	-	3	2	23	1	30	26
-	-	3910	-	-	-	4	3	23	2	26	26
-	-	1748	-	-	-	5	4	23	3	26	30
-	-	2085	-	-	-	6	5	23	4	26	30
-	-	-	-	-	-	7	6	23	5	26	30
-	-	-	-	-	-	8	1	23	1	26	30
-	-	-	-	-	-	9	2	23	2	26	30
-	-	-	-	-	-	1	3	22	3	26	30
-	-	-	-	-	-	2	4	22	4	26	30
-	-	-	-	-	-	3	5	22	1	26	30
-	-	-	-	-	-	1	6	22	2	26	30
-	-	-	-	-	-	2	7	22	3	-	30
-	-	-	-	-	-	3	1	21	4	-	30
-	-	-	-	-	-	1	2	21	1	-	27
-	-	-	-	-	-	2	3	21	1	-	-
-	-	-	-	-	-	3	1	21	-	-	-
-	-	-	-	-	-	2	2	21	-	-	-
-	-	-	-	-	-	3	3	21	-	-	-
-	-	-	-	-	-	4	4	21	-	-	-
-	-	-	-	-	-	5	5	21	-	-	-
-	-	-	-	-	-	1	6	21	-	-	-
-	-	-	-	-	-	2	1	21	-	-	-
-	-	-	-	-	-	3	2	-	-	-	-
-	-	-	-	-	-	-	3	-	-	-	-
-	-	-	-	-	-	-	4	-	-	-	-
-	-	-	-	-	-	-	1	-	-	-	-
-	-	-	-	-	-	-	2	-	-	-	-
-	-	-	-	-	-	-	3	-	-	-	-
-	-	-	-	-	-	-	4	-	-	-	-
-	-	-	-	-	-	-	5	-	-	-	-
-	-	-	-	-	-	-	1	-	-	-	-
-	-	-	-	-	-	-	2	-	-	-	-
-	-	-	-	-	-	-	3	-	-	-	-

-	-	-	-	-	-	-	4	-	-	-	-
-	-	-	-	-	-	-	1	-	-	-	-
-	-	-	-	-	-	-	2	-	-	-	-
-	-	-	-	-	-	-	3	-	-	-	-
-	-	-	-	-	-	-	4	-	-	-	-
-	-	-	-	-	-	-	5	-	-	-	-
-	-	-	-	-	-	-	6	-	-	-	-
